# Supplementary material for: Modulating multi-functional ERK complexes by covalent targeting of a recruitment site in vivo
Source: Nat Commun. 2019 Nov 19;10:5232. doi: 10.1038/s41467-019-12996-8 (PMC6863825; doi:10.1038/s41467-019-12996-8)
Supplement: Supplementary file 1 — Supplementary Information [file 41467_2019_12996_MOESM1_ESM.pdf]

Supplementary Information

Modulating multi-functional ERK complexes by covalent targeting of a recruitment site *in vivo*

Kaoud *et al*.

Supplementary Figures, Tables and Notes:

a.

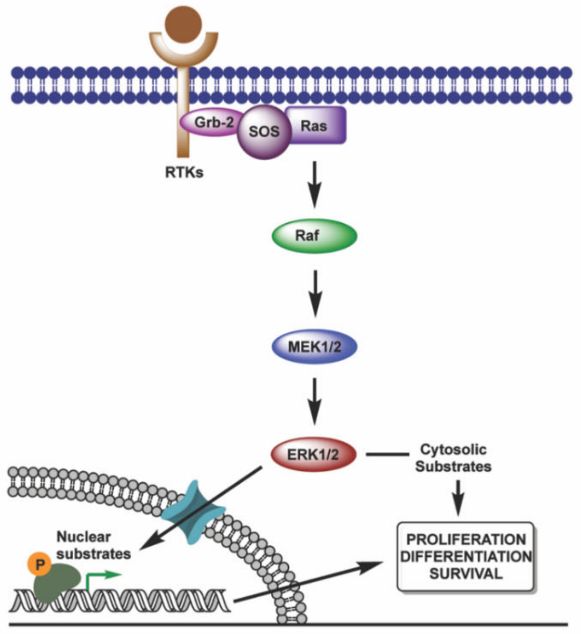

b.

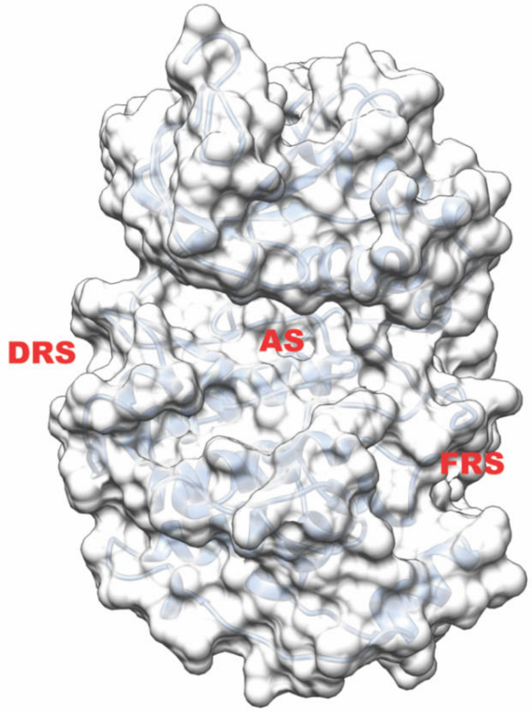

Supplementary Fig. 1. a The Raf/MEK/ERK pathway. **b** A surface representation of ERK2 showing the D-recruitment site (DRS), the F-recruitment site (FRS) and the active site (AS).

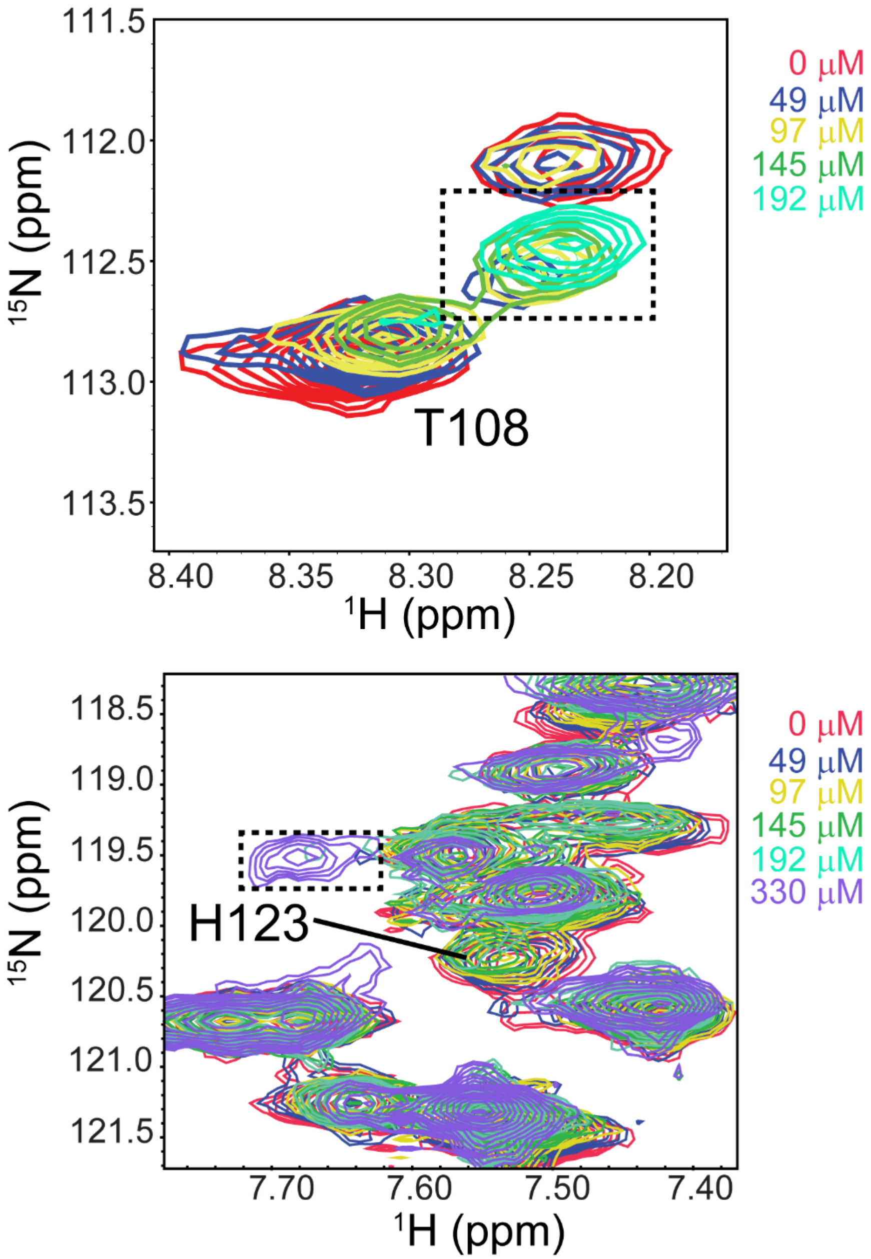

Supplementary Fig. 2 Examples of new peaks (enclosed by the dashed rectangle) appearing due to the slow exchange regime during the titration course with BI-78D3 in the ^{15}N, ^{1}H TROSY spectra of ERK2 (600 MHz). Expansions of the spectrum centered near the T108 (top panel) and H123 (bottom panel) resonances are shown. Samples contained 200 μM ERK2.

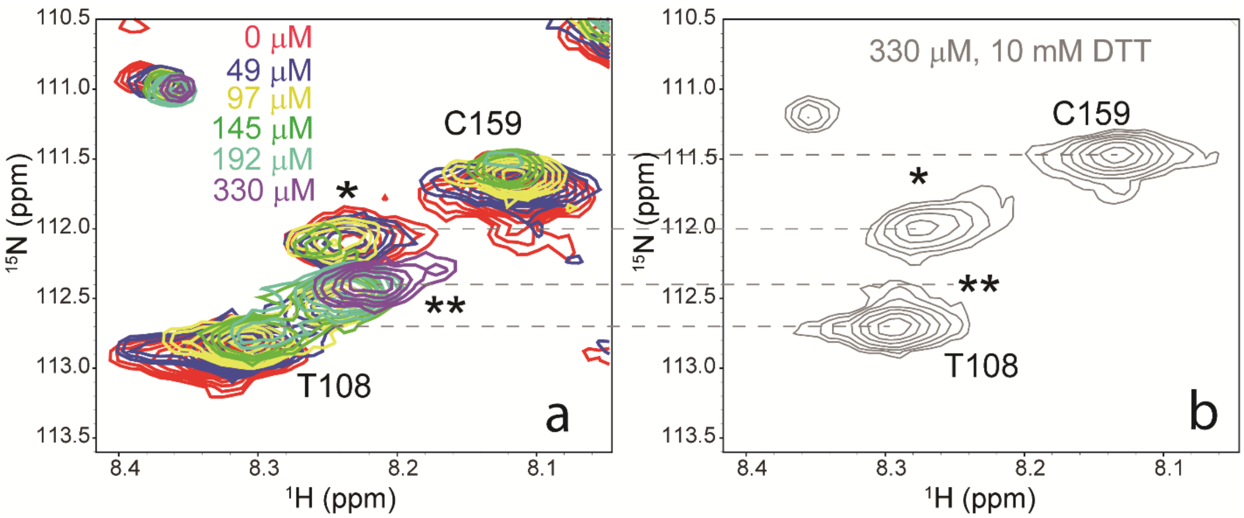

Supplementary Fig. 3 a Progressive reduction in the intensities of the resonances corresponding to T108 and C159 in free ERK2 upon addition of an increasing amount of BI-78D3 (also see Fig. 1c). The positions of the peaks that are quenched also experience a small shift as a result of increased presence of DMSO (the solvent for BI-78D3). A similar overall effect is seen for an adjacent yet unassigned peak marked '*'. Also note the appearance of a new peak marked '**' corresponding to T108 in the C159-modified form of ERK2 that appears during the course of the titration (also see Supplementary Fig. 2 above for further examples of the appearance of new peaks due to the "slow exchange" regime). **b** Addition of 10 mM DTT at the end of the titration course leads to the reappearance of the peaks corresponding to T108, C159 and the peak marked '*' in free ERK2. The slightly shifted new positions of these peaks are consistent with the direction of the shifts seen in (a) due to the presence of DMSO. It is notable that the peak marked '**' (corresponding to T108 in BI-78D3-modified ERK2) disappears completely upon addition of DTT. Samples contained 200 μM ERK2.

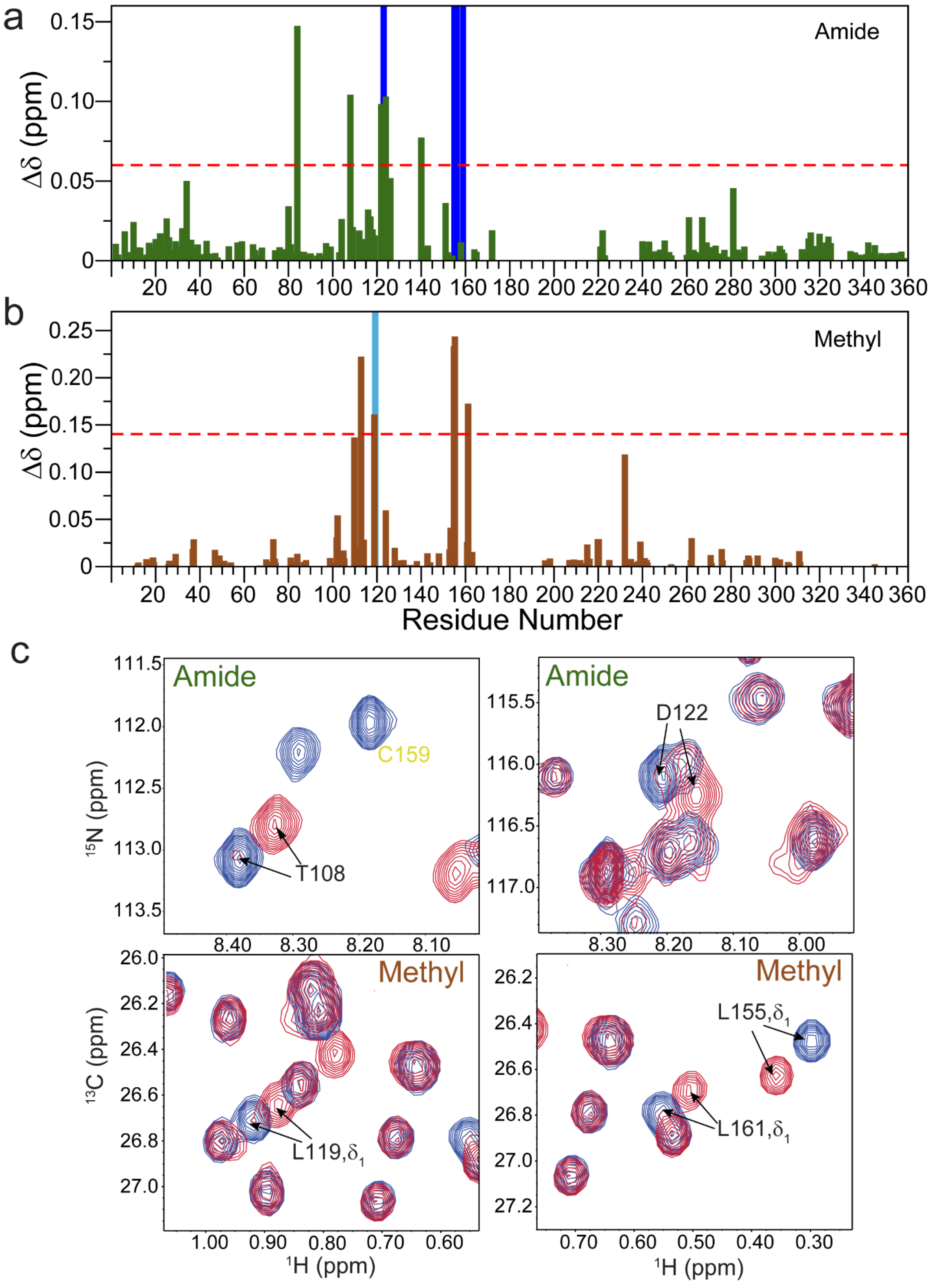
a
Amide
Δδ (ppm)
0.15
0.10
0.05
0
20 40 60 80 100 120 140 160 180 200 220 240 260 280 300 320 340 360
b
Methyl
Δδ (ppm)
0.25
0.20
0.15
0.10
0.05
0
Residue Number
c
Amide
C159
T108
^{15}N (ppm)
D122
Methyl
L119,δ1
L155,δ1
L161,δ1
^{13}C (ppm)
^{1}H (ppm)

Supplementary Fig. 4 Chemical shift perturbations (Δδ) for **a** backbone amide ^{15}N, ^{1}H or **b** Ile, Leu and Val methyl ^{13}C, ^{1}H resonances on inactive ERK2 (116 μM) in the presence of an approximately equimolar amount of BI-78D3 (130 μM). The Δδ values are indicated by green and brown bars for the amide and methyl resonances, respectively. Amide (including C159) and methyl resonances that are broadened out to below the noise are indicated by blue and cyan bars, respectively. The red dashed line indicates $\Delta\delta + 2.5\sigma = 0.06$ ppm for amides and 0.14 for methyl resonances, respectively. **c** Representative ^{15}N, ^{1}H TROSY (top panel) and ^{13}C, ^{1}H HMQC spectra (bottom panel) both acquired at 800 MHz for ERK2 alone (blue) and bound to BI-78D3 (red). The resonance positions of selected perturbed residues are labeled.

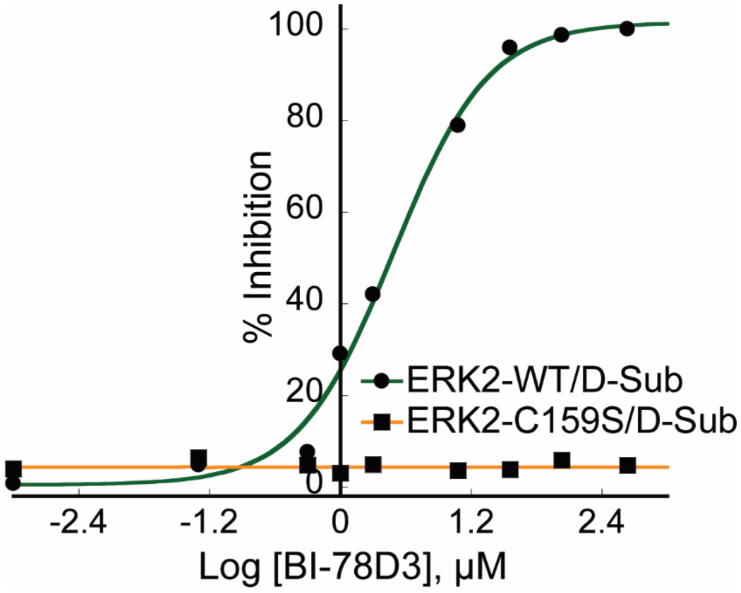

Supplementary Fig. 5 Evaluating the ability of BI-78D3 to inhibit ERK2 and ERK2 C159S**.** Different concentrations of BI-78D3 were incubated with activated ERK2 WT and ERK2 C159S for 30 minutes, before addition of D-sub peptide and [γ- ^{32}P] ATP. (Data represents one experiment out of two repetitions).

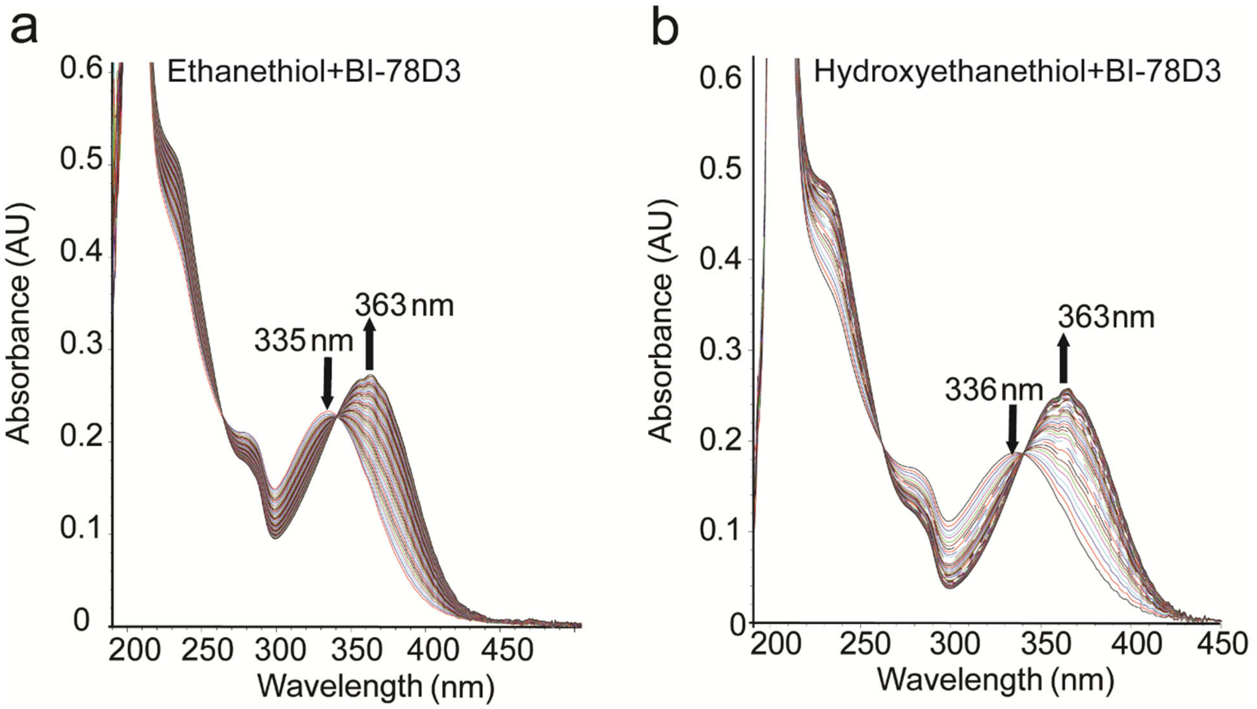

Supplementary Fig. 6 Observed change in UV-visible spectrum for the reaction of **a** ethanethiol (100 μM) or **b** hydroxyethanethiol (100 μM) with BI-78D3 (10 μM) in 50 mM phosphate buffer, pH 7.5 and 2% dioxane (spectra recorded every 10 seconds for 1000 second).

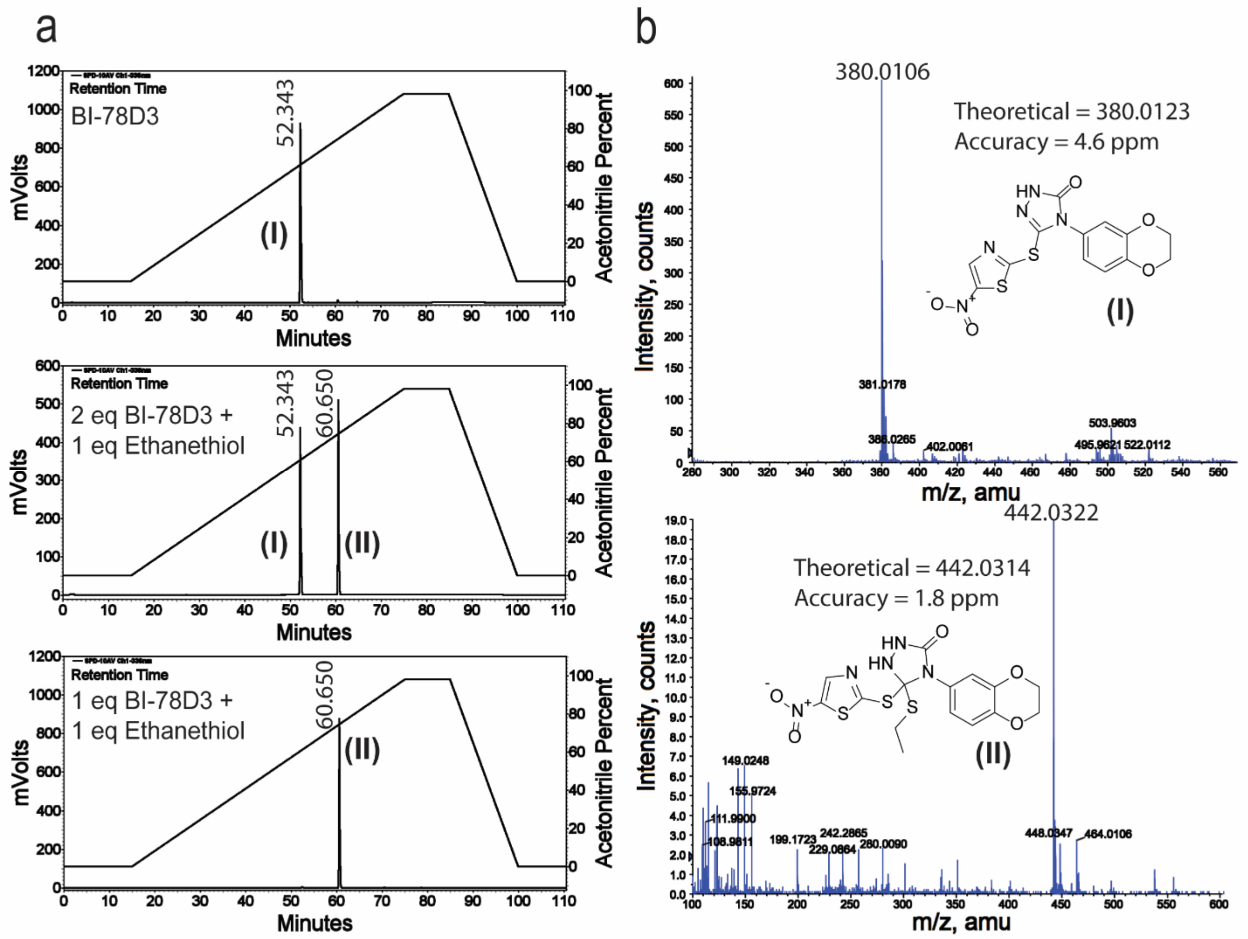

Supplementary Fig. 7 a HPLC analysis of the reaction between BI-78D3 and ethanethiol. Peak (I) [t_R ~ 52 min] corresponds to BI-78D3. Peak (II) [t_R ~ 60 min] corresponds to the product of the reaction. **b** High resolution mass spectra (HRMS) of HPLC fractions corresponding to peak (I) (observed 380.0106 Da, theoretical 380.0123 Da) and peak (II) (observed 442.0322 Da, theoretical 442.0314 Da). Reaction conditions: BI-78D3 (20 µL of a 50 mM solution in DMSO) was mixed with a solution of ethanethiol (10 or 20 µL of a 50 mM solution in DMSO), volume completed to 50 µL DMSO and mixed with 50 µL phosphate buffer (50 mM pH 7.5). The reaction was continued for 60 minutes at room temperature then 25 µL of this reaction was mixed with 200 µL of water before injection into the HPLC system. HPLC conditions Altima 5 µ C18 column at 25 °C and a flow rate of 3 mL min^{-1}, absorbance wavelengths 336 and 360 nm; Mobile phase solvent A was

water and solvent B was acetonitrile. HPLC fractions were flash frozen instantly, until directly injected into the HRMS system.

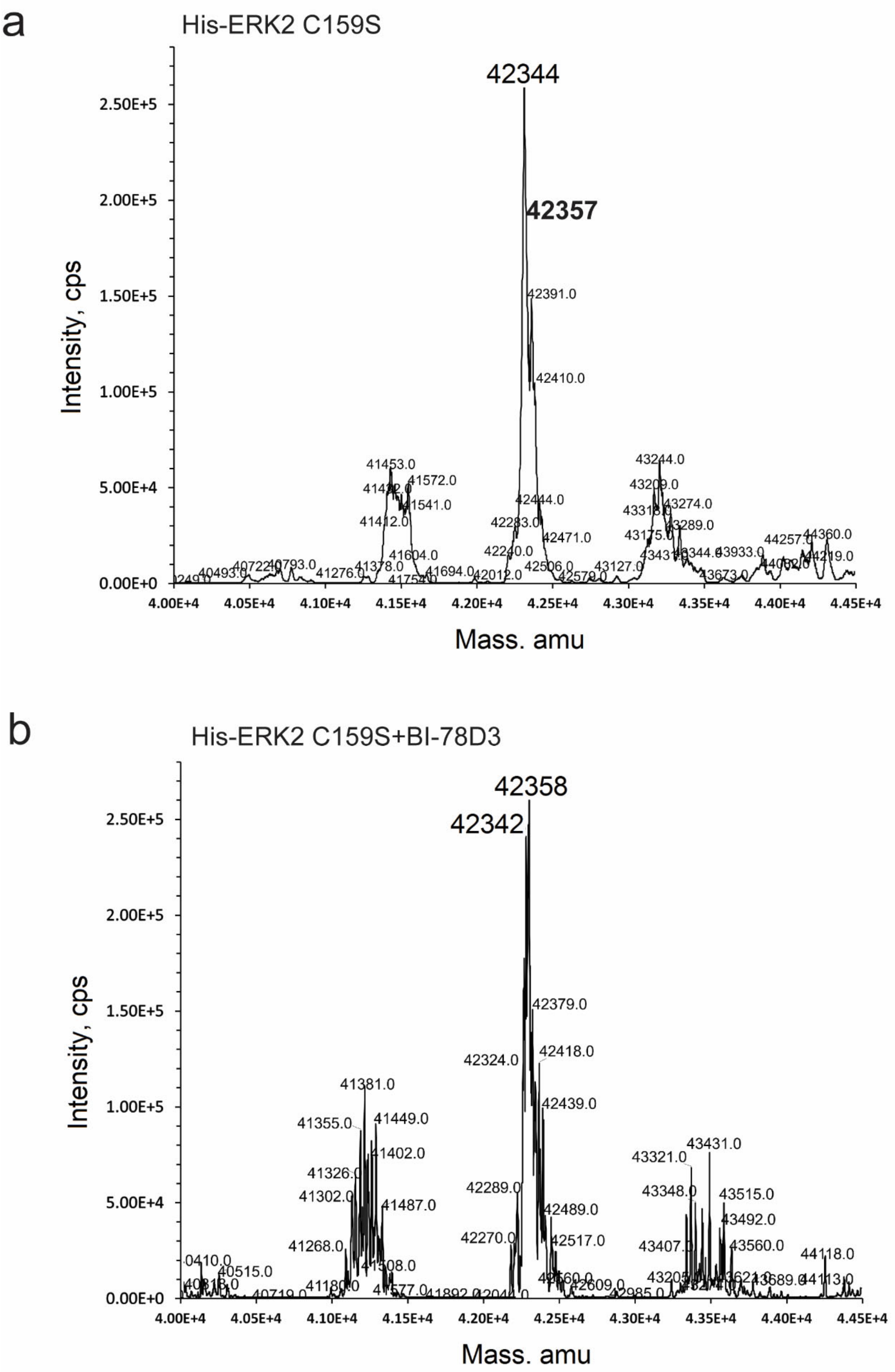
a
His-ERK2 C159S
42344
42357
42391.0
42410.0
41453.0
41572.0
41541.0
41412.0
41604.0
41694.0
41378.0
41276.0
40793.0
40493.0
42444.0
42471.0
42240.0
42012.0
42506.0
43244.0
43274.0
43289.0
43175.0
43127.0
43933.0
43673.0
44257.0
44360.0
Intensity, cps
Mass. amu
0.00E+0
5.00E+4
1.00E+5
1.50E+5
2.00E+5
2.50E+5
4.00E+4
4.05E+4
4.10E+4
4.15E+4
4.20E+4
4.25E+4
4.30E+4
4.35E+4
4.40E+4
4.45E+4
b
His-ERK2 C159S+BI-78D3
42358
42342
42379.0
42324.0
42418.0
42439.0
42289.0
42489.0
42270.0
42517.0
42609.0
42985.0
41381.0
41355.0
41449.0
41402.0
41326.0
41302.0
41487.0
41268.0
41508.0
40515.0
40719.0
43431.0
43321.0
43348.0
43515.0
43492.0
43560.0
43407.0
44118.0
Intensity, cps
Mass. amu
0.00E+0
5.00E+4
1.00E+5
1.50E+5
2.00E+5
2.50E+5
4.00E+4
4.05E+4
4.10E+4
4.15E+4
4.20E+4
4.25E+4
4.30E+4
4.35E+4
4.40E+4
4.45E+4

Supplementary Fig. 8 Mass spectrometry analysis of active His-ERK2 C159S following incubation with BI-78D3. 5 μM recombinant ERK2 (C159S mutant) was incubated with **a** DMSO or **b** 100 μM BI-78D3 for 160 minutes, followed by buffer exchange using a PD-10 desalting column.

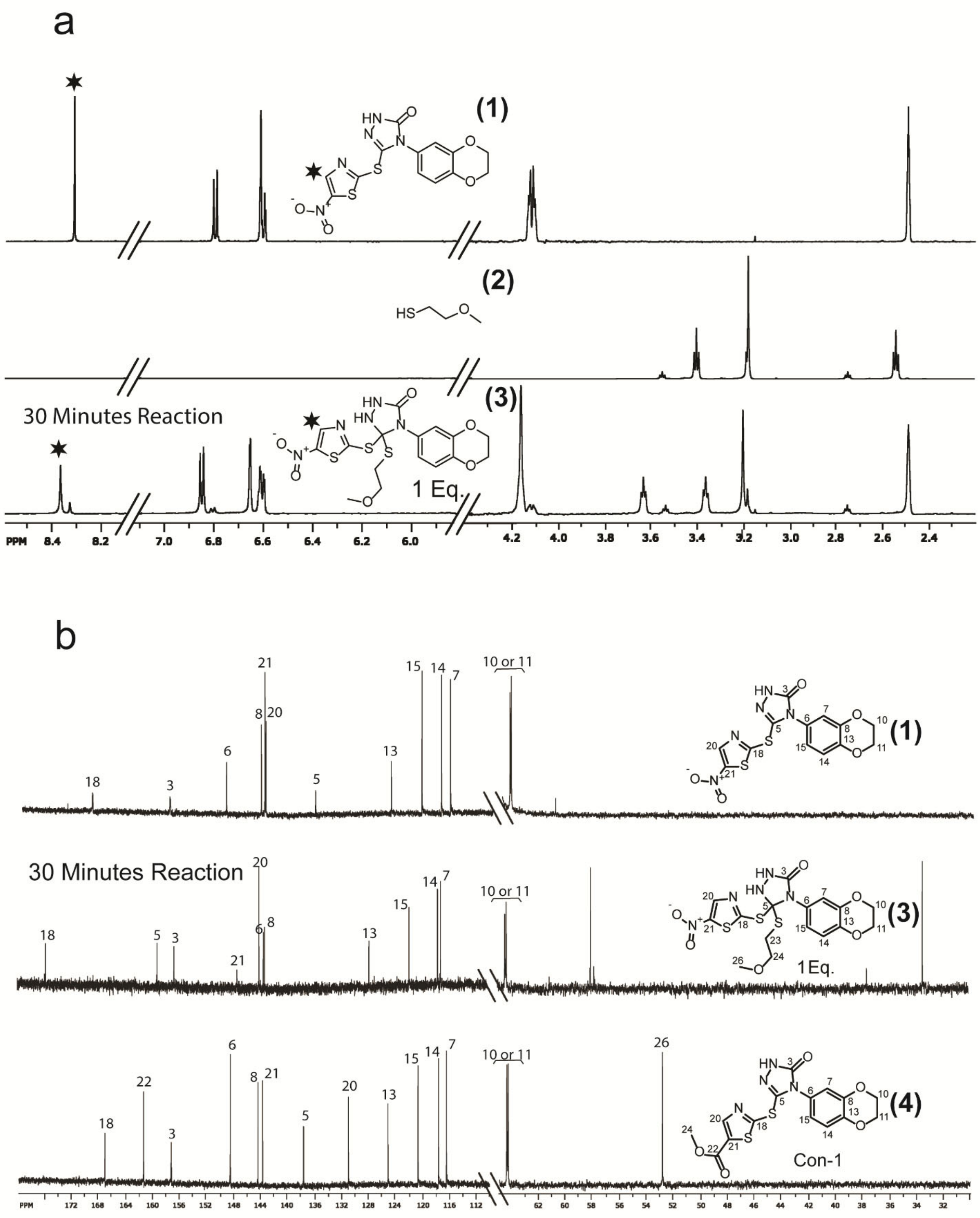

Supplementary Fig. 9 a ^{1}H NMR spectrum (600 MHz) of **(1)** BI-78D3, **(2)** 2-methoxyethanethiol and **(3)** the product of the reaction between equimolar **(1)** and **(2)**. To an NMR tube was added 300

μL Dioxane-d_8 containing 4 mg of **(1)**. The reaction was initiated at room temperature by the addition of 300 μL of 50 mM phosphate buffer, pH 7.5 containing an equimolar amount of **(2)**. The spectrum was acquired in array (every 3 minutes) after initiation of the reaction (30 minutes spectrum is shown). **b** ^{13}C NMR spectrum (500 MHz in ^{1}H frequency, Bruker Avance III equipped with cryogenic probes) of **(1)** BI78D3, **(3)** the product of the reaction between **(1)** and 2-methoxyethanethiol **(2)** with equal molarity (same reaction conditions as above) and an authentic sample of **(4)** Con-1 (supplementary methods, synthesis). To an NMR tube was added 300 μL Dioxane-d_8 containing 4 mg of **(1)**. The reaction was initiated at room temperature by the addition of 300 μL of 50 mM phosphate buffer, pH 7.5 containing an equal molar amount of **(2)**. We started to scan the sample within 2-3 minutes after initiation of the reaction, 512 scans were acquired in a total of 29 minutes.

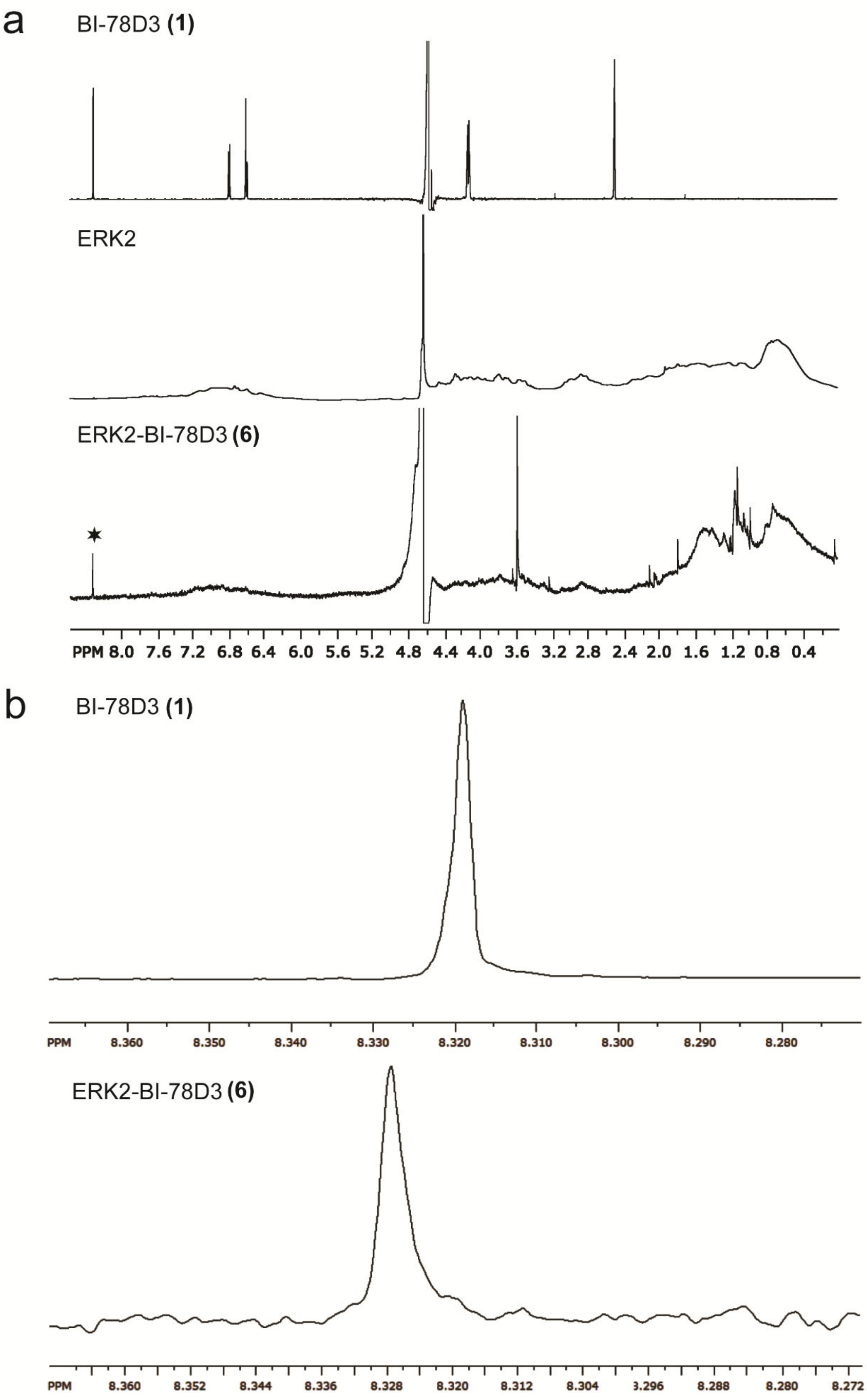
a
BI-78D3 (1)
ERK2
ERK2-BI-78D3 (6)
PPM 8.0 7.6 7.2 6.8 6.4 6.0 5.6 5.2 4.8 4.4 4.0 3.6 3.2 2.8 2.4 2.0 1.6 1.2 0.8 0.4
b
BI-78D3 (1)
PPM 8.360 8.350 8.340 8.330 8.320 8.310 8.300 8.290 8.280
ERK2-BI-78D3 (6)
PPM 8.360 8.352 8.344 8.336 8.328 8.320 8.312 8.304 3.296 8.288 8.280 8.272

Supplementary Fig. 10 a ^{1}H NMR spectrum (600 MHz, recorded on Varian DirectDrive 600 MHz spectrometer (Palo Alto, CA)) of **(1)**, non-phosphorylated His-ERK2 and **(6)** the product of the reaction between **(1)** and ERK2. 11 mg of inactive His tagged ERK2 were allowed to react with BI-78D3 in 50 mM phosphate buffer, pH 7.5 and 5% dioxane for 15-20 minutes at room temperature, the labeled protein was desalted using three PD 10 columns, buffer was exchanged for 6 times to phosphate D2O buffer solution of pH 7.5 using Amicon concentrators (Millipore). Each time the buffer was diluted 1/2 in deuterated buffer without dioxane, and so some water and dioxane was still present (partial exchange of water and dioxane) and the spectrum was acquired immediately. The experiment was repeated three times and the results were consistent. 100 % labeling of ERK2 by BI-78D3 was confirmed by ESI-MS and UV-Vis spectrophotometry. The asterisks indicate the C4 proton of the thiazole ring. **b** The resonance marked by the asterisk in the upper figure was expanded, the proton peak at 8.33 ppm of the ERK2-BI-78D3 **(6)** is still broader (full width at half maximum ~ 2.0 Hz) and shifted downfield if compared to the same peak in an authentic sample of BI-78D3 (full width at half maximum ~ 1.2 Hz) **(1)**.

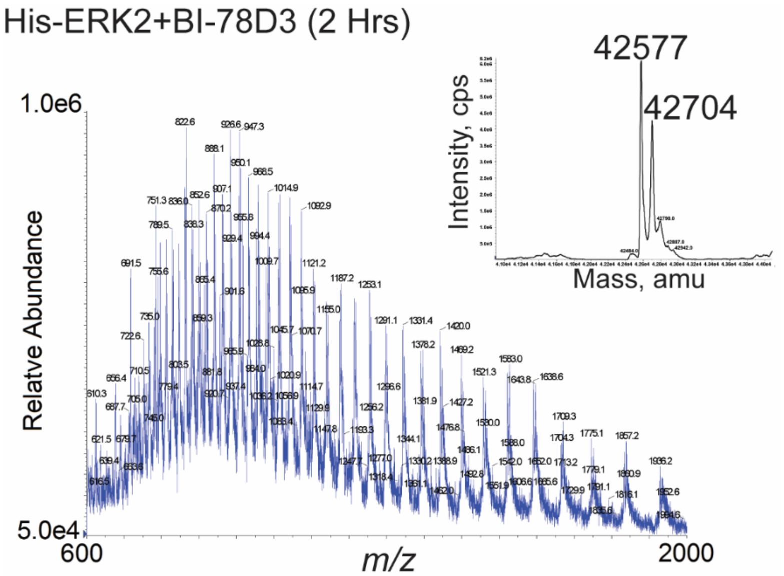

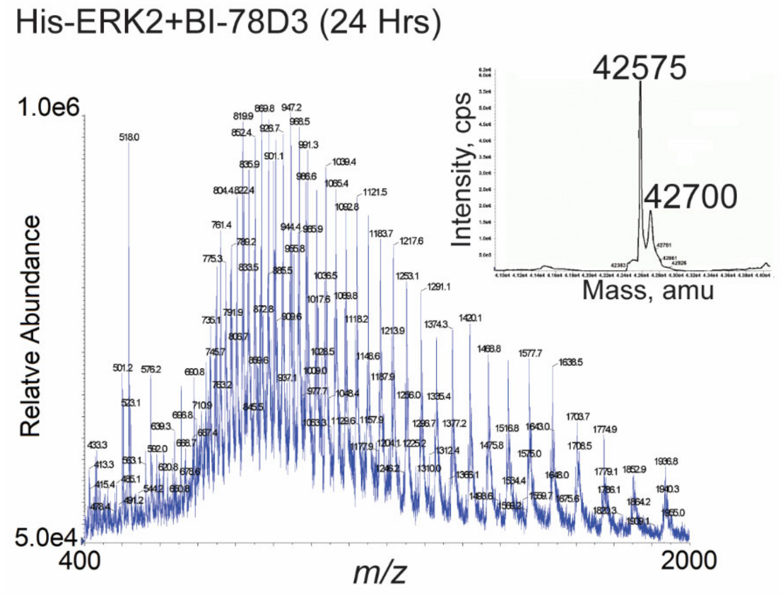

Supplementary Fig. 11 Deconvoluted mass spectra of ERK2 showing the mass change after incubation of ERK2 (5 μM) with BI-78D3 (100 μM) for 2 or 24 Hours, followed by buffer exchange using a PD-10 column. (Calculated molecular weights of BI-78D3 and ERK2 are 380 and 42329 Da. respectively).

Supplementary Note 1 –Mutational analysis of recombinant ERK2

We mutated a number of residues that exhibited significant perturbations in the NMR spectra of ERK2 upon adduct formation, to alanine. These included L155, N156, and C159 of loop 11, T108 of the inter-lobe linker, H123 of helix αE, D316 and D319 of the Φchg pocket and C164 of the active site. Each mutant was expressed, purified, and activated (see the Methods section) and shown to possess Michaelis-Menten parameters using a peptide substrate, indistinguishable from that of the wild type enzyme (Supplementary Table 1). As expected, mutations at the Φchg pocket (D316A and D319A) and in the active site (C164A) had no effect on the susceptibility of ERK2 to BI-78D3 (determined as an apparent IC_{50}). Similarly, L155A, H123A were not significantly distinguishable from the wild type protein. However, mutation of C159S completely abrogated the ability of BI-78D3 to inhibit and the T108A and N156A mutants showed a 3-to-4-fold increase in the apparent IC_{50} (Supplementary Table 1).

Supplementary Table 1: The effect of different ERK2 DRS mutations on the sensitivity of ERK2 towards BI-78D3.

**Mutants**	**IC_{50} (μM)***	**K_m (μM)****	**k_{cat} (s^{-1})****	**k_{cat}/K_m ($\mu M^{-1}\ s^{-1}$)**
T108A	13.12±1.3	18.7±2.3	29.9±1.2	1.60
H123A	5.1±1.0	22.7±3.9	16.5±1.0	0.73
L155A	4.5±0.8	14.5±1.6	36.9±1.3	2.54
N156A	11.1±1.3	18.7±3.7	35.9±2.3	1.91
C159S	No inhib	12.8±1.9	17.3±0.8	1.35
C164A	3.1±0.6	9.30±0.9	19.6±0.5	2.10
D316A	4.4±0.7	20.7±5.0	17.4±1.4	0.84
D319A	5.4±0.8	17.2±1.9	12.1±0.4	0.70
WT	3.0±0.4	10.2±1.4	23.0±0.9	2.25

*Detected by measuring the ability of each mutant to phosphorylate the D-sub peptide in the presence of different concentrations of BI-78D3. Different concentrations of BI-78D3 were incubated with activated ERK2 mutants or WT for 30 minutes before addition of D-sub peptide and [γ- ^{32}P] ATP.

** Effect of ERK2 mutations on its Michaelis–Menten kinetic parameters using Ets-1 as a protein substrate.

Supplementary Note 2 – The reactivity of BI-78D3 towards other proteins

Upon incubation of BI-78D3 with recombinant ERK2, p38-α MAPK, ERK5, JNK1, JNK2, JNK3, and JNK2 C163A (C163 corresponds to C159 in ERK2) which were expressed, purified and stored in 50 mM phosphate buffer (pH 7.5) containing 10% glycerol. ERK2 was the only protein that showed a characteristic change in the absorption spectrum, consistent with thiol addition (Supplementary Fig. 12). On the other hand, reaction of DNTB with each tested protein revealed one or more surface accessible cysteines (Supplementary Table 2). Interestingly, mutation of C163 of JNK2 to alanine did not impact the reactivity of the protein with DNTB, suggesting that C163 is probably not accessible to DNTB. Additionally, we used LC-MS to assess whether other MAPKs could form an adduct following incubation with BI-78D3 (10 μM) for 60 minutes. We could not detect the labeling of either His-JNK2 (Supplementary Fig. 13a), p38-α MAPK (Supplementary Fig. 13c) or ERK5 (Supplementary Fig. 13b).

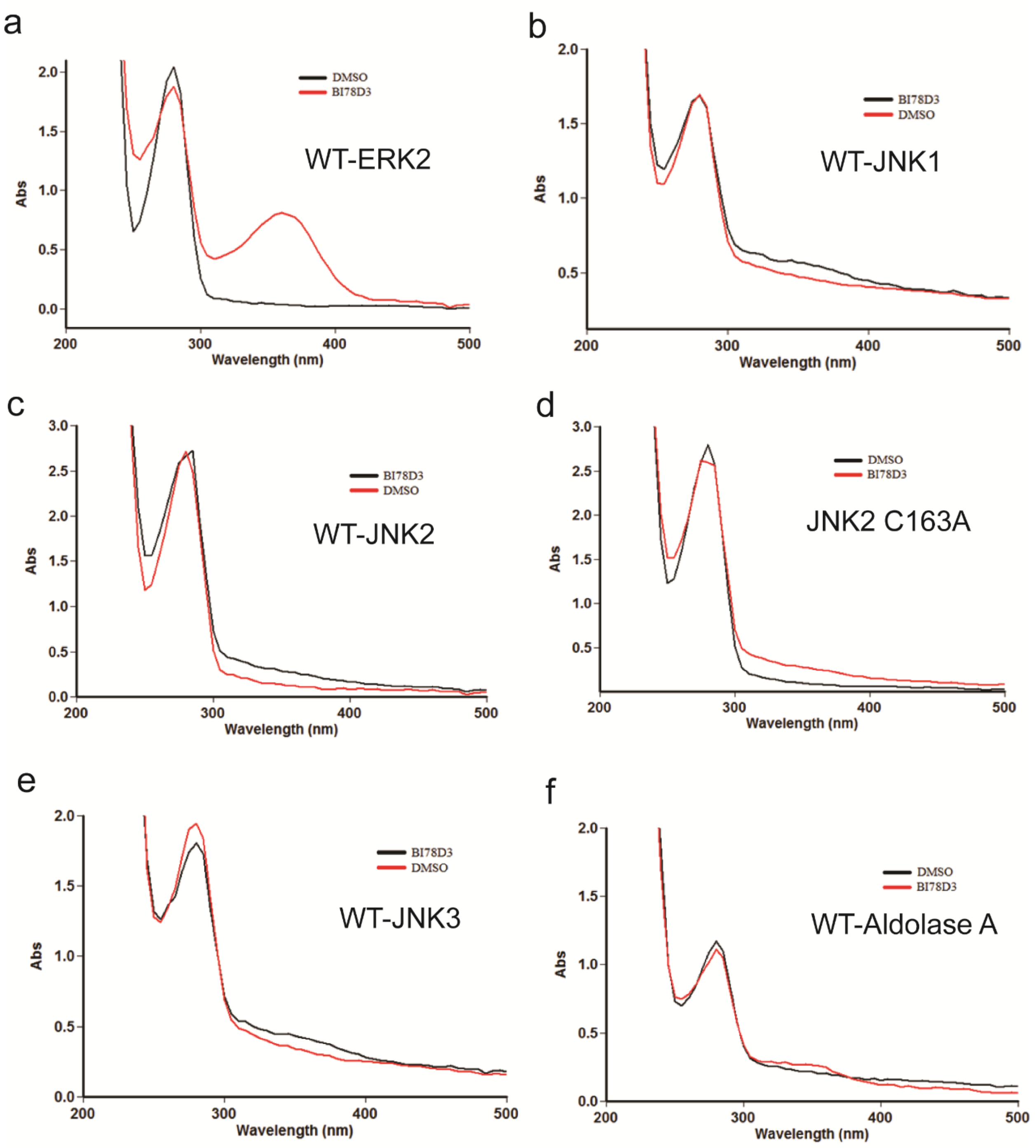

Supplementary Fig. 12 Assessment of the reactivity of BI-78D3 towards **a** WT-ERK2, **b** JNK1, **c** JNK2, **d** JNK2 C163A, **e** JNK3, and **f** Aldolase A. In brief, DMSO or 100 µM of BI78D3 were allowed to react with 5 µM of each enzyme for 60 minutes. The reaction mixture was dialyzed overnight in phosphate buffer containing 10% glycerol (two dilutions each 1/4000). Proteins were

concentrated back to 50 μM and the UV spectra of the dialyzed proteins were recorded to evaluate the ability of each protein to form a covalent adduct with BI-78D3 (more details are available in the methods section). WT-ERK2 was the only enzyme that showed a characteristic change in the absorption spectrum, consistent with thiol addition.

Supplementary Table 2: The number of free cysteines in each protein does not correlate with the ability of each protein to react with BI-78D3

Protein Tested	Average Surface Cysteines*	±SD (n=3)	Reported Cysteines	UV spectrum change with BI-78D3**
ERK2	3.6	0.359	7 Cysteines<br>GeneBank Accession NM_053842	Yes
JNK1	1.4	0.1	8 Cysteines<br>GeneBank Accession NM_002750	No
JNK2	0.7	0.05	10 Cysteines<br>GeneBank Accession NM_002752	No
JNK2-C163A	0.7	0.01	9 Cysteines	No
JNK3	1	0.06	11 Cysteines<br>GeneBank Accession NM_138982	No
p38alpha	3.0	0.117	4 Cysteines<br>GeneBank Accession NM_011951.3	No
ERK5	1.9	0.383	4 Cysteines (aa 31-391)<br>GeneBank Accession NP_002740.2	No
BSA	0.4	0.060	35 Cysteines, only one is free.[1]	No
Esterase	1.6	0.123	6 Cysteines, only two are free.[2]	No
Aldolase A	1.5	0.11	3 free Cysteines, one reacts very fast with DTNB and two very slow.[3]	No
Lysozyme	0.1	0.095	8 Cysteines, all in disulfide bonds[4]	No

*Were estimated in triplicate using Ellman's reagent (pierce) and the native proteins, following the manufacture protocol.

** UV spectra of 50 µM enzyme reacting with 10 µM BI-78D3 in 50 mM phosphate buffer (pH 7.5) containing 2% dioxane were recorded every 10 seconds over 600 seconds on an Agilent 8453 diode-array spectrophotometer.

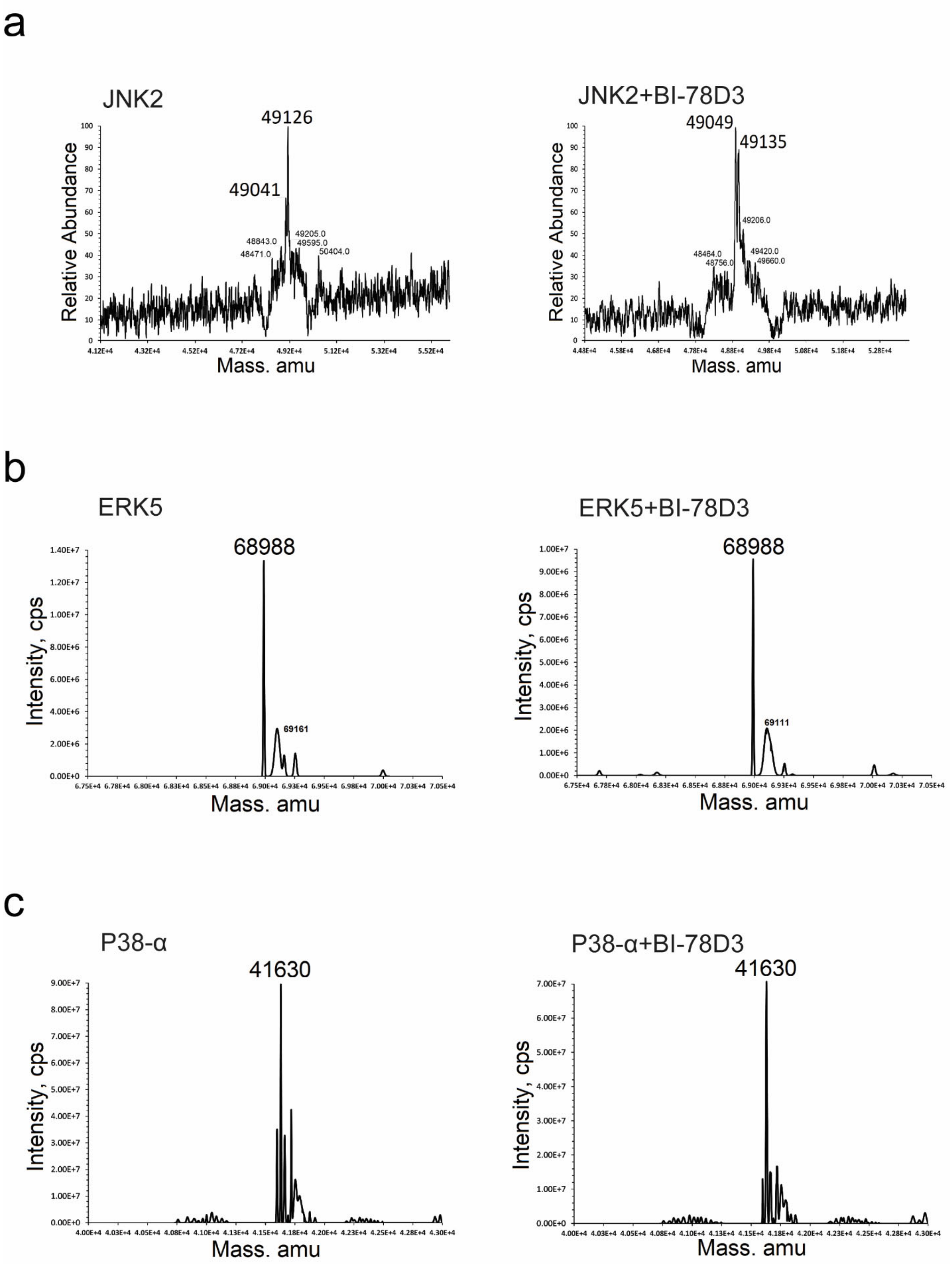
a
JNK2
49126
49041
48843.0
48471.0
49205.0
49595.0
50404.0
Relative Abundance
Mass. amu
JNK2+BI-78D3
49049
49135
49206.0
48464.0
48756.0
49420.0
49660.0
Relative Abundance
Mass. amu
b
ERK5
68988
69161
Intensity, cps
Mass. amu
ERK5+BI-78D3
68988
69111
Intensity, cps
Mass. amu
c
P38-α
41630
Intensity, cps
Mass. amu
P38-α+BI-78D3
41630
Intensity, cps
Mass. amu

Supplementary Fig. 13 Assessment of the reactivity of BI-78D3 towards **a** full length JNK2, **b** ERK5 catalytic domain and **c** full length P38-α by mass spectrometry. Recombinant kinases (5 μM) were incubated with DMSO, or BI-78D3 (100 μM) for 60 minutes. Excess compound was removed using a PD-10 desalting column. A deconvoluted mass spectrum for each kinase was produced using ESI-MS.

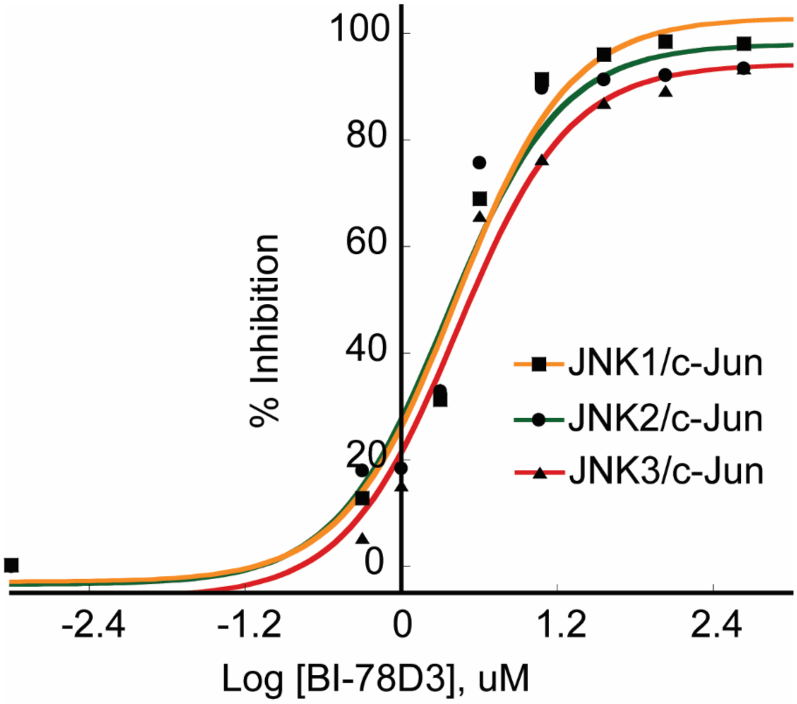

Supplementary Fig. 14 Assessing the inhibition of recombinant JNK kinases by BI-78D3. Different concentrations of BI-78D3 were incubated with JNK1, JNK2 or JNK3 for 60 minutes, before the addition of c-JUN and [γ- ^{32}P] ATP. (Data represents one experiment out of two repetitions)

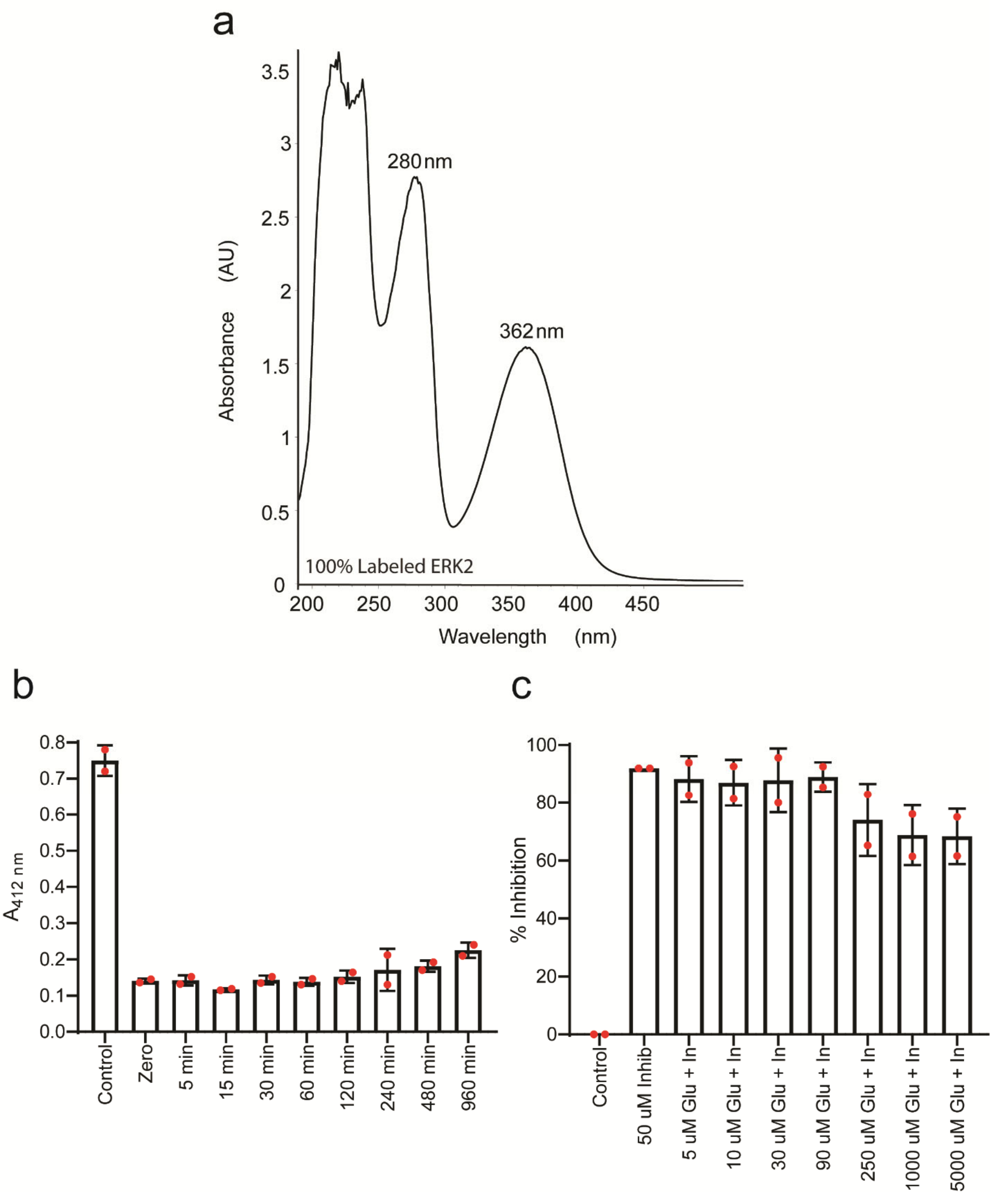
a
Absorbance (AU)
3.5
3
2.5
2
1.5
1
0.5
0
280 nm
362 nm
100% Labeled ERK2
200
250
300
350
400
450
Wavelength (nm)
b
$A_{412\ nm}$
0.8
0.7
0.6
0.5
0.4
0.3
0.2
0.1
0.0
Control
Zero
5 min
15 min
30 min
60 min
120 min
240 min
480 min
960 min
c
% Inhibition
100
80
60
40
20
0
Control
50 uM Inhib
5 uM Glu + In
10 uM Glu + In
30 uM Glu + In
90 uM Glu + In
250 uM Glu + In
1000 uM Glu + In
5000 uM Glu + In

Supplementary Fig. 15 a UV-Vis spectrum of an adduct (50 μM) formed between His-ERK2 and BI-78D3 in 50 mM phosphate buffer, pH 7.5. **b** Assessment of adduct stability using Ellman's reagent. The reactivity of the adduct formed between BI-78D3 and ERK2, bearing only one cysteine at C159, towards Ellman's reagent was assessed for 16 hours (More details in the methods section) (Data are from 2 independent experiments, and bars represent mean ± (SD) standard deviation). **c** Assessment of the reactivity of the ERK•BI-78D3 adduct with glutathione. BI-78D3 (50 μM) was incubated with activated ERK2 for 30 minutes before dilution in a reaction mixture containing different concentrations of glutathione (0-5000 μM), 10 μM D-Sub peptide substrate and 500 μM [γ- 32P] ATP. ERK2 incubated with 5% DMSO was employed as a control. (Data are from 2 independent experiments, and bars represent mean ± SD).

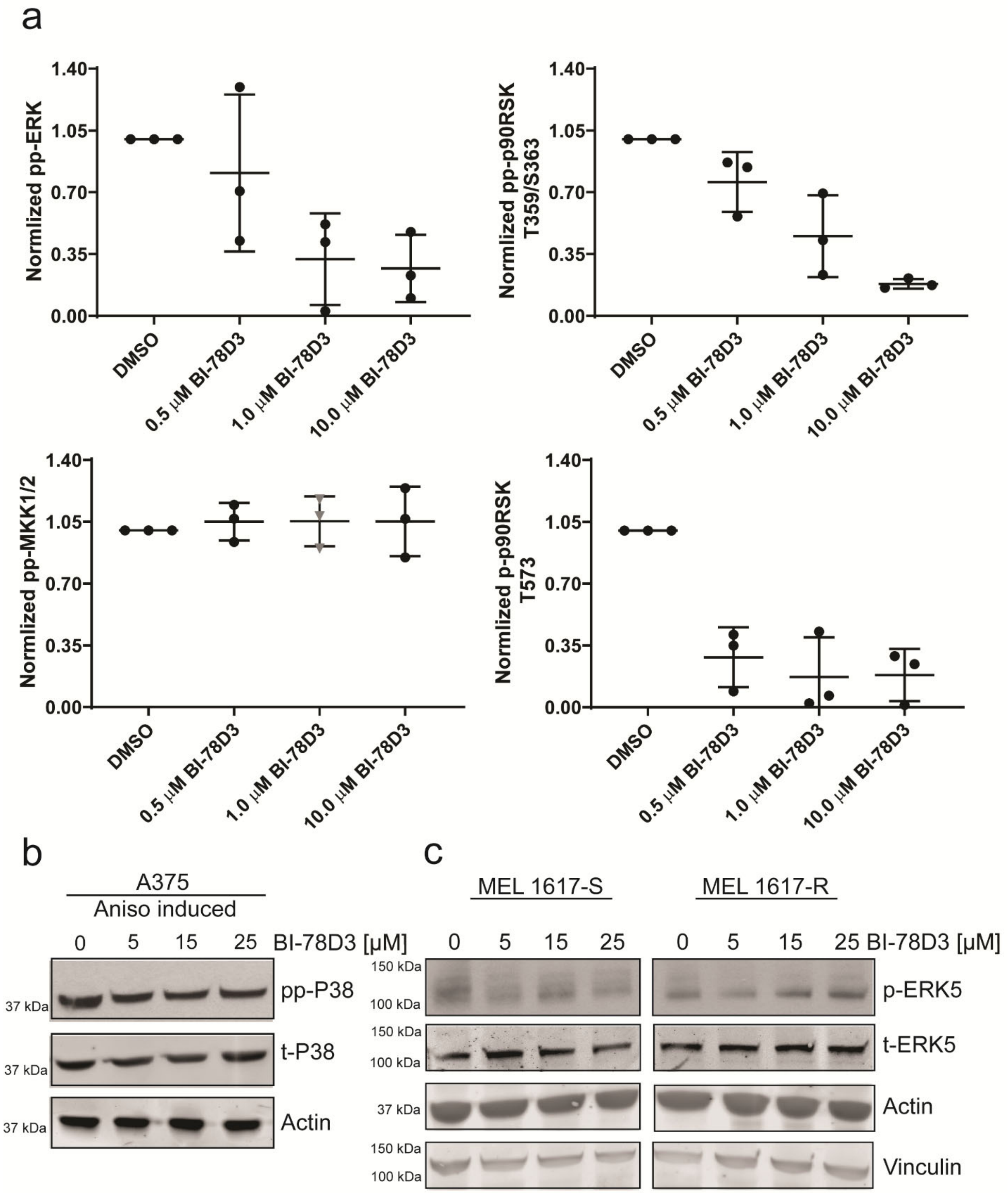

Supplementary Fig. 16 a Quantification of the effect of BI-78D3 on phosphorylation of ERK, p90RSK and MKK1/2 in *BRAF*-mutant A375 melanoma cells. The densitometric analysis of the Immunoblots was performed using NIH imageJ Fiji software.[5] These data represent the average of

three different experiments, the uncropped immunoblots are shown in Supplementary Figs. 25 and 26. Each phosphoprotein signal was normalized to the corresponding total protein signal. In this experiment, 1.5 million cells were seeded in 60 mm dish, serum starved overnight, treated with BI-78D3 for 1 hour in serum free media, followed by inhibitor washout for 2 hours in the same media. Cells were then stimulated with EGF for 30 minutes in full media. Detailed protocols of **c**ell lysis and immunoblotting are mentioned in the methods section. **b** BI-78D3 did not show remarkable inhibition of P38-α phosphorylation in A375 cell line. Cells were serum starved overnight then treated with different doses of BI-78D3 (0–25 μM) for 60-90 minutes, followed by 15 minutes stimulation with anisomycin before cell lysis. Images have been cropped for presentation, uncropped images are shown in Supplementary Fig. 27. **c** BI-78D3 did not inhibit ERK5 phosphorylation in PLX4032-sensitive (MEL 1617-S) or resistant (MEL 1617-R), *BRAF*-mutant melanoma cell line MEL 1617. Cells were serum starved overnight then treated with different doses of BI-78D3 (0–25 μM) for 60-90 minutes, followed by 15 minutes stimulation with EGF before cell lysis. Images have been cropped for presentation, uncropped images are shown in Supplementary Fig. 27.

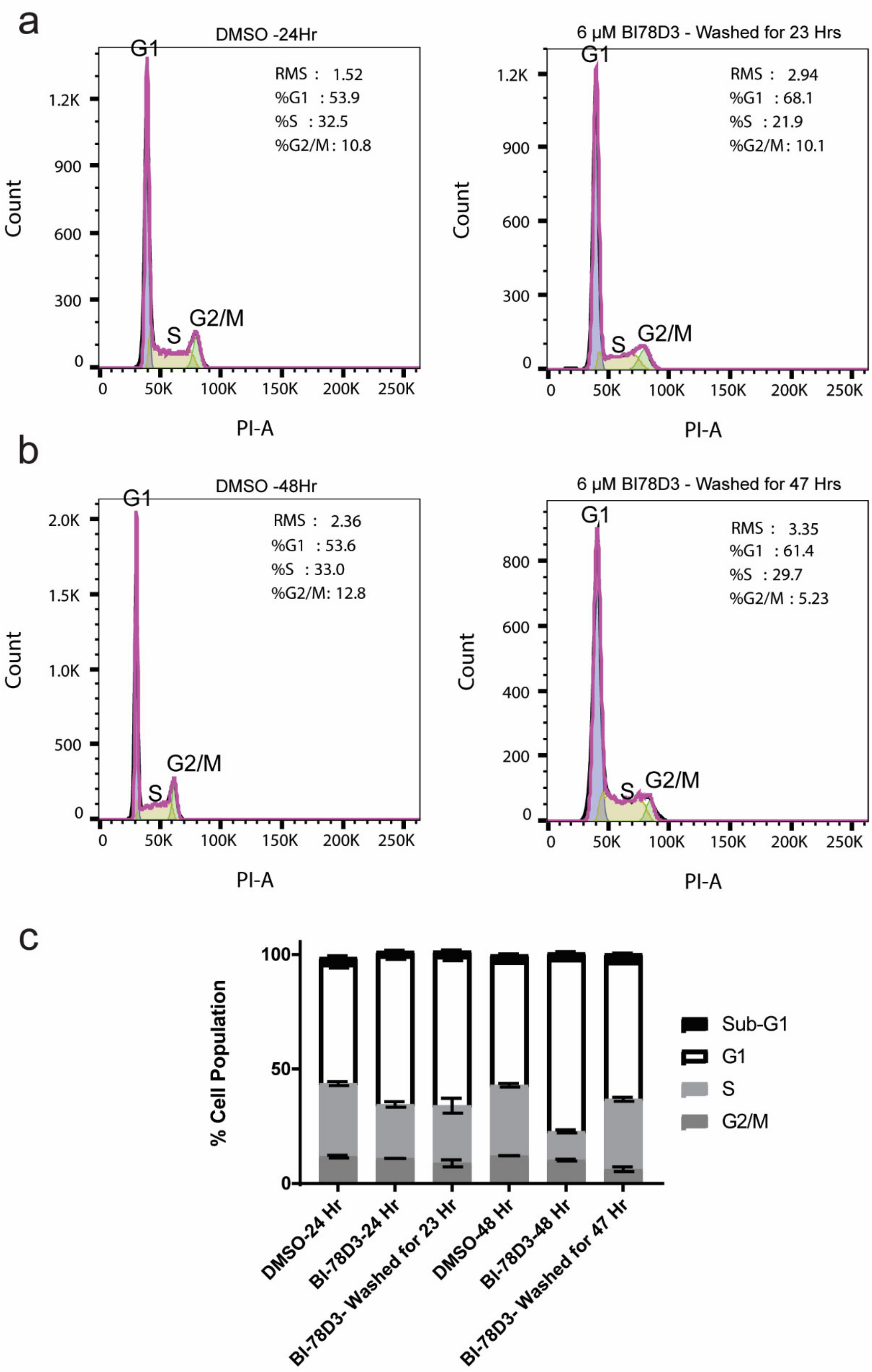
a
DMSO -24Hr
G1
RMS : 1.52
%G1 : 53.9
%S : 32.5
%G2/M : 10.8
S
G2/M
Count
PI-A
6 μM BI78D3 - Washed for 23 Hrs
RMS : 2.94
%G1 : 68.1
%S : 21.9
%G2/M : 10.1
b
DMSO -48Hr
RMS : 2.36
%G1 : 53.6
%S : 33.0
%G2/M: 12.8
6 μM BI78D3 - Washed for 47 Hrs
RMS : 3.35
%G1 : 61.4
%S : 29.7
%G2/M : 5.23
c
% Cell Population
Sub-G1
G1
S
G2/M
DMSO-24 Hr
BI-78D3-24 Hr
BI-78D3- Washed for 23 Hr
DMSO-48 Hr
BI-78D3-48 Hr
BI-78D3- Washed for 47 Hr

Supplementary Fig. 17 FACS analysis of PI-stained A375 cell nuclei after treatment with either 6 μM of BI-78D3 or DMSO control for 24 **a** and 48 **b** hours. Cells were washed by PBS after 1 hour of BI-78D3 or DMSO treatment, then incubated in fresh full media for the rest of the experiment (23 and 47 hours respectively). The PI fluorescence was analyzed using the modeling feature of FlowJo V10 following the previously published model by Watson et al[6]. In all cases the % of cells modelled in each phase was comparable. RMS is root mean square deviation, represents the goodness of the fit of the model to the data. **c** Histogram showing the percentages of cells in each phase of the cell cycle for both experiments where BI-78D3 was washed after 1 hour of treatment or incubated with the cells for the entire experiment (An average of three independent experiments, Error bars SEM).

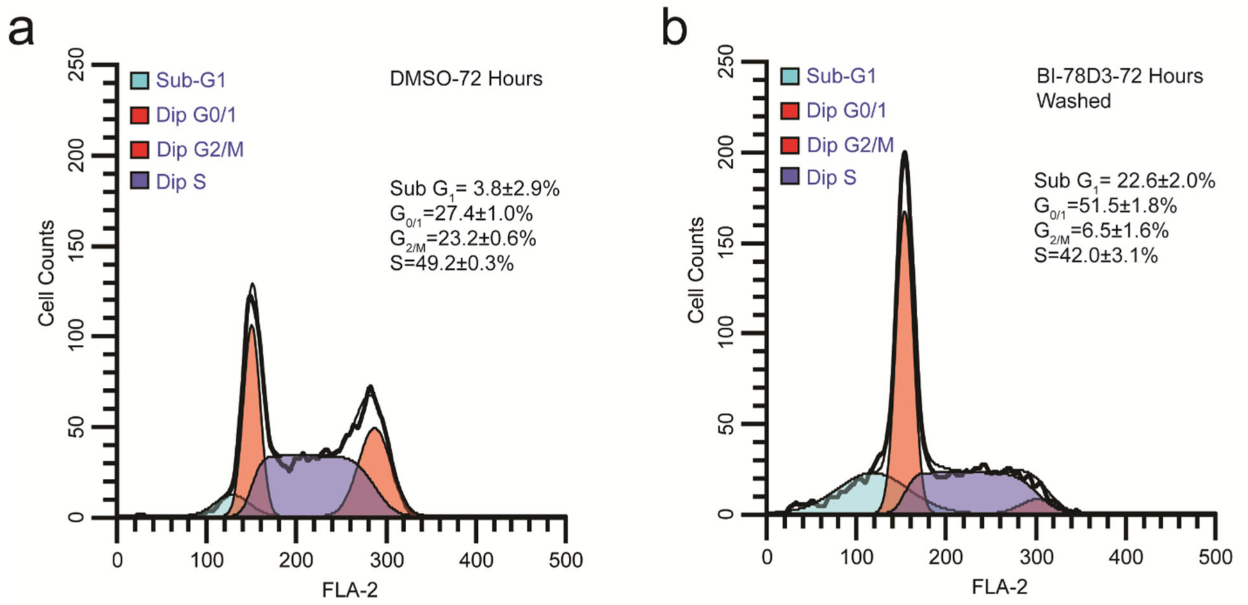

Supplementary Fig. 18 FACS analysis of A375 cell stained with propodeum iodide after treatment with **a** control vehicle (DMSO) or **b** 6 µM BI-78D3 for 72 hours. Cells were washed by PBS after 1 hour of treatment, then incubated in fresh full media for the rest of the experiment. The percentage of cells in apoptosis (Sub-G1 population) were estimated using the ModFit 3.0 software. (The experiments have been repeated at least four times).

Supplementary Note 3 – Chemical genetics validate Cys-159 as a site of vulnerability to BI-78D3 in melanoma cells

To investigate whether the vulnerability of A375 cells to BI-78D3 can be attributed to the suppression of ERK docking through the DRS, we employed a chemical genetics approach. Transient expression of ERK2 C159A mutant (an inhibitor-resistant form of ERK2) in A375 cells rescued the ability of BI-78D3 to suppress ERK signaling (Supplementary Fig. 19), ERK nuclear localization (Supplementary Fig. 20), and both anchorage-independent (Supplementary Fig. 21a) and dependent (Supplementary Fig. 21b) colonies formation. The transient over-expression of wild type ERK also blunted the effect of BI-78D3, but the effect was smaller. The same observations were seen in HEK 293 cells (Supplementary Fig. 22). This suggests that BI-78D3 suppresses cell proliferation and survival through covalent binding to C159 of ERK.

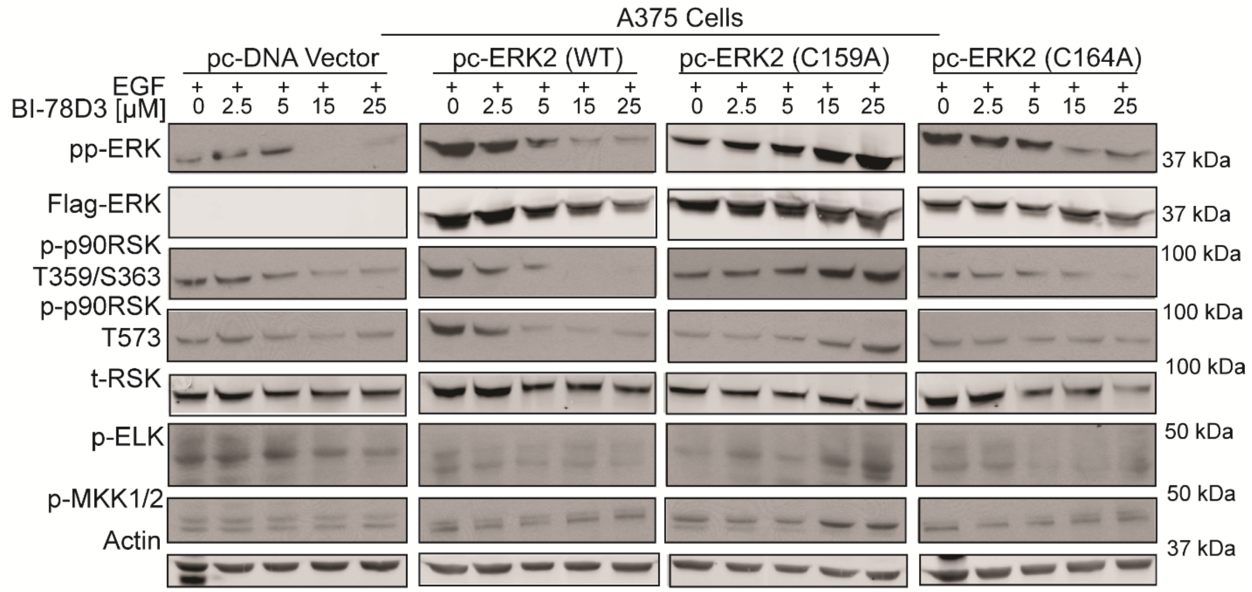

Supplementary Fig. 19 Altering ERK2 sensitivity to BI-78D3 in *BRAF*-mutant A375 cell lines. Cells over-expressing empty pcDNA vector, Flag-ERK2 (WT), Flag-ERK2 (C159A) or Flag-ERK2 (C164A) were serum starved overnight then treated with different doses of BI-78D3 (0–25 μM) for 60-90 minutes before induction by EGF. Subsequent phosphorylation of ERK, p90RSK, ELK and MEK1/2 was detected by western blot. C159A mutant, but not C164A mutant, rescued ERK inhibition by BI-78D3. Images have been cropped for presentation, uncropped images are shown in Supplementary Fig. 28.

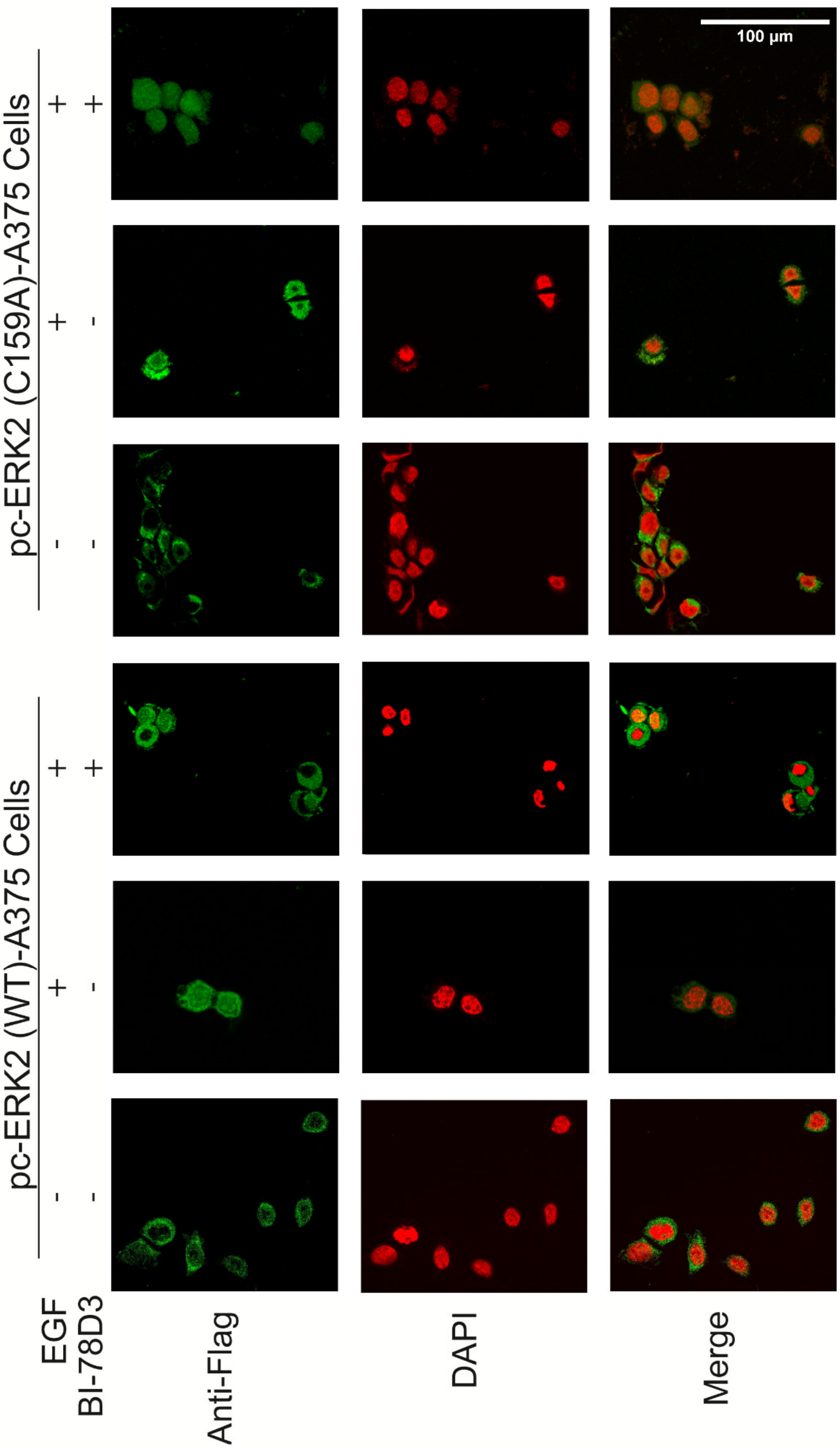
pc-ERK2 (WT)-A375 Cells
pc-ERK2 (C159A)-A375 Cells
EGF
BI-78D3
- - + - + +
- - + - + +
Anti-Flag
DAPI
Merge
100 μm

Supplementary Fig. 20 Rendering transiently transfected ERK2 refractive to BI-78D3 in A375 cells suppresses inhibition of EGF-stimulated nuclear translocation by BI-78D3. Transfected A375 cells were serum starved overnight, then treated with 25 μM of BI-78D3 for 1 hour and induced with EGF. Cells were fixed, immune-stained and imaged using a confocal microscope. Scale bar represents 100 μm.

a

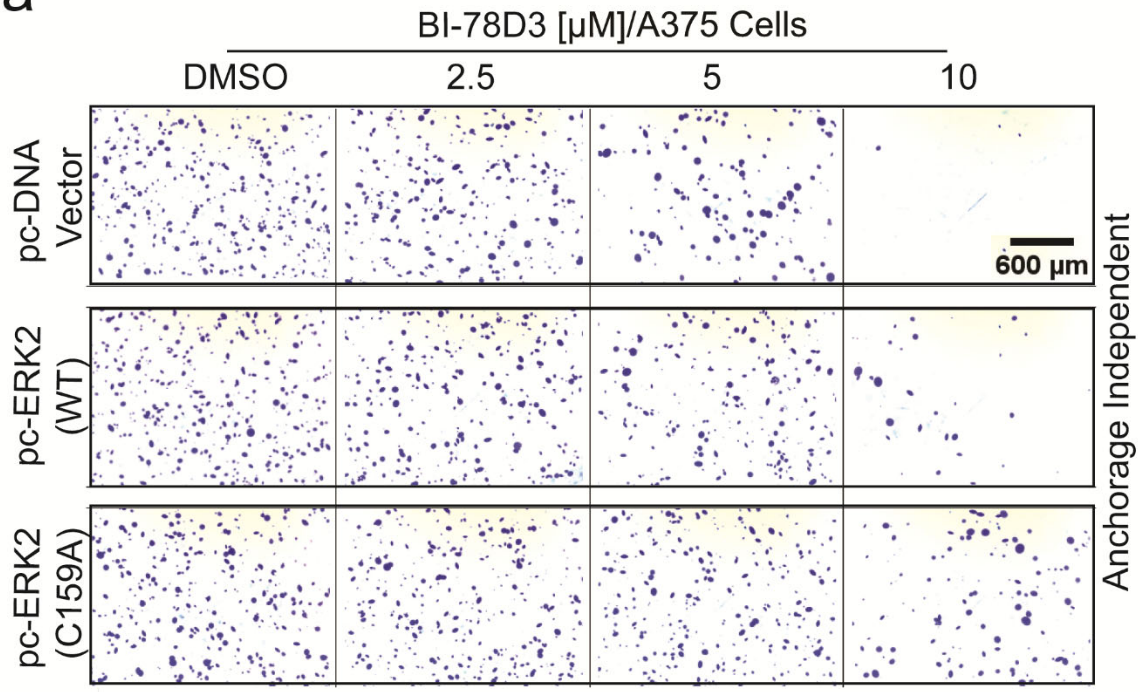
BI-78D3 [μM]/A375 Cells
DMSO
2.5
5
10
pc-DNA Vector
pc-ERK2 (WT)
pc-ERK2 (C159A)
600 μm
Anchorage Independent

b

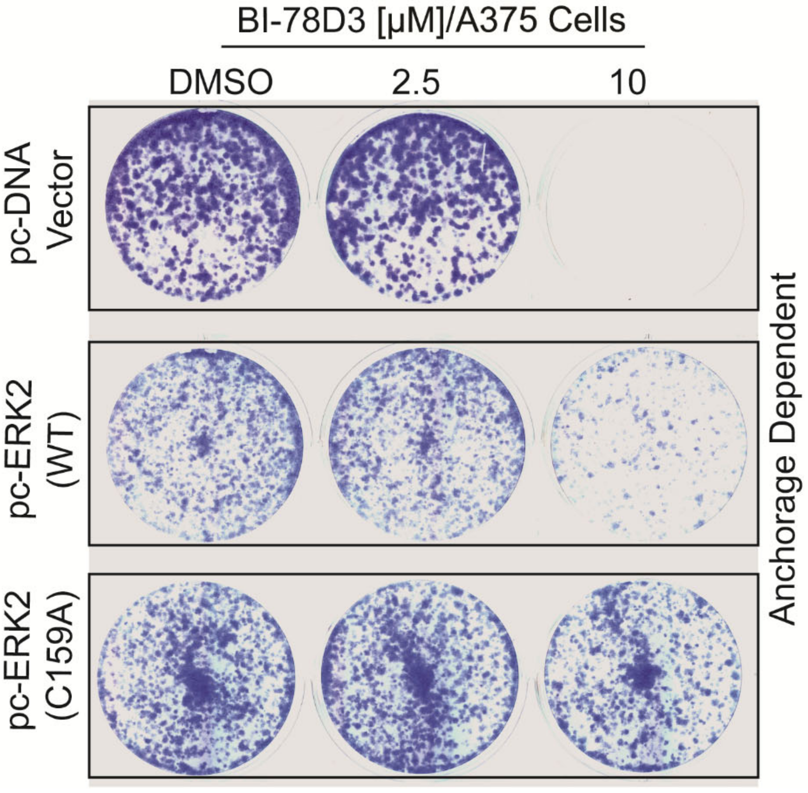
BI-78D3 [μM]/A375 Cells
DMSO
2.5
10
pc-DNA Vector
pc-ERK2 (WT)
pc-ERK2 (C159A)
Anchorage Dependent

Supplementary Fig. 21: Altering ERK2 sensitivity to BI-78D3 in A375. Overexpression of ERK2-C159A in A375 cells rescues the inhibitory effect of BI-78D3 on **a** anchorage-independent and **b** -dependent growth. Cells overexpressing empty pc-DNA and ERK2-WT-pc-DNA vectors were employed as a control. A375 cells over-expressing empty pcDNA vector, flag-ERK2 (WT) or flag-ERK2 (C159A), were treated with different doses of BI-78D3 (0–10 μM). (The experiments have been repeated two times). Scale bar represents 600 μm.

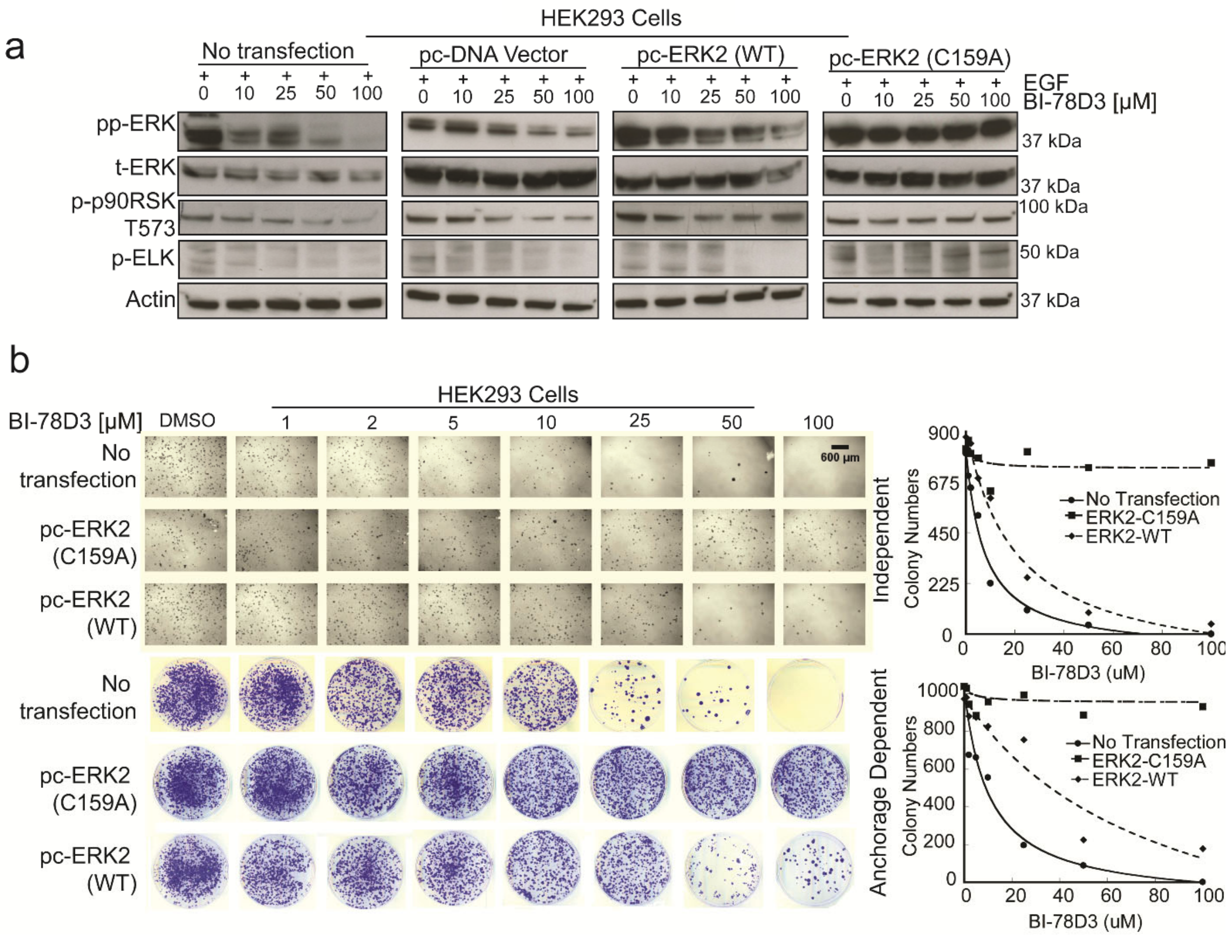

Supplementary Fig. 22 Altering ERK sensitivity to BI-78D3 in HEK293 cells **a** HEK293 cells were transfected with empty pc-DNA vector, pc-DNA ERK2 (WT) or pc-DNA ERK2 (C159A), serum starved overnight then treated with different doses of BI-78D3 (0–100 µM) for 120 minutes before induction by EGF. Subsequent phosphorylation of ERK, p90RSK and ELK was detected by western blot. Images have been cropped for presentation, uncropped imaged are shown in Supplementary Fig. 29. **b** BI-78D3 inhibited the anchorage-dependent and independent growth of HEK293 cells not transfected or transfected with pcDNA-ERK2 (WT) in a dose dependent manner. Scale bar represents 600 µm. Transfection of pcDNA-ERK2 (C159A), rescued the dose-dependent inhibition colony formation. The colonies number for each experiment were quantified

using Open CFU software[7] and plotted using Kaleidagraph software. (The Colonies data are from 2 independent experiments).

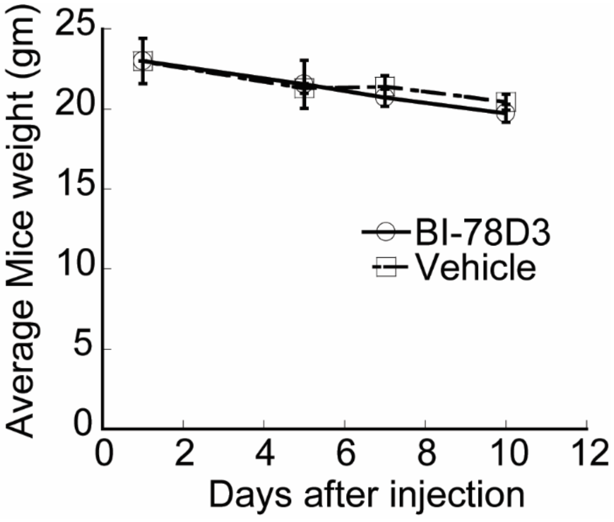

Supplementary Fig. 23 Average body weights of BI-78D3 treated mice relative to vehicle control animals in A375 xenograft. 15 mg kg^{-1} daily dose of BI-78D3 did not show any significant lethality over the 10 days treatment period, if compared to the control mice. The values plotted as Mean ± SD (n=10 per group).

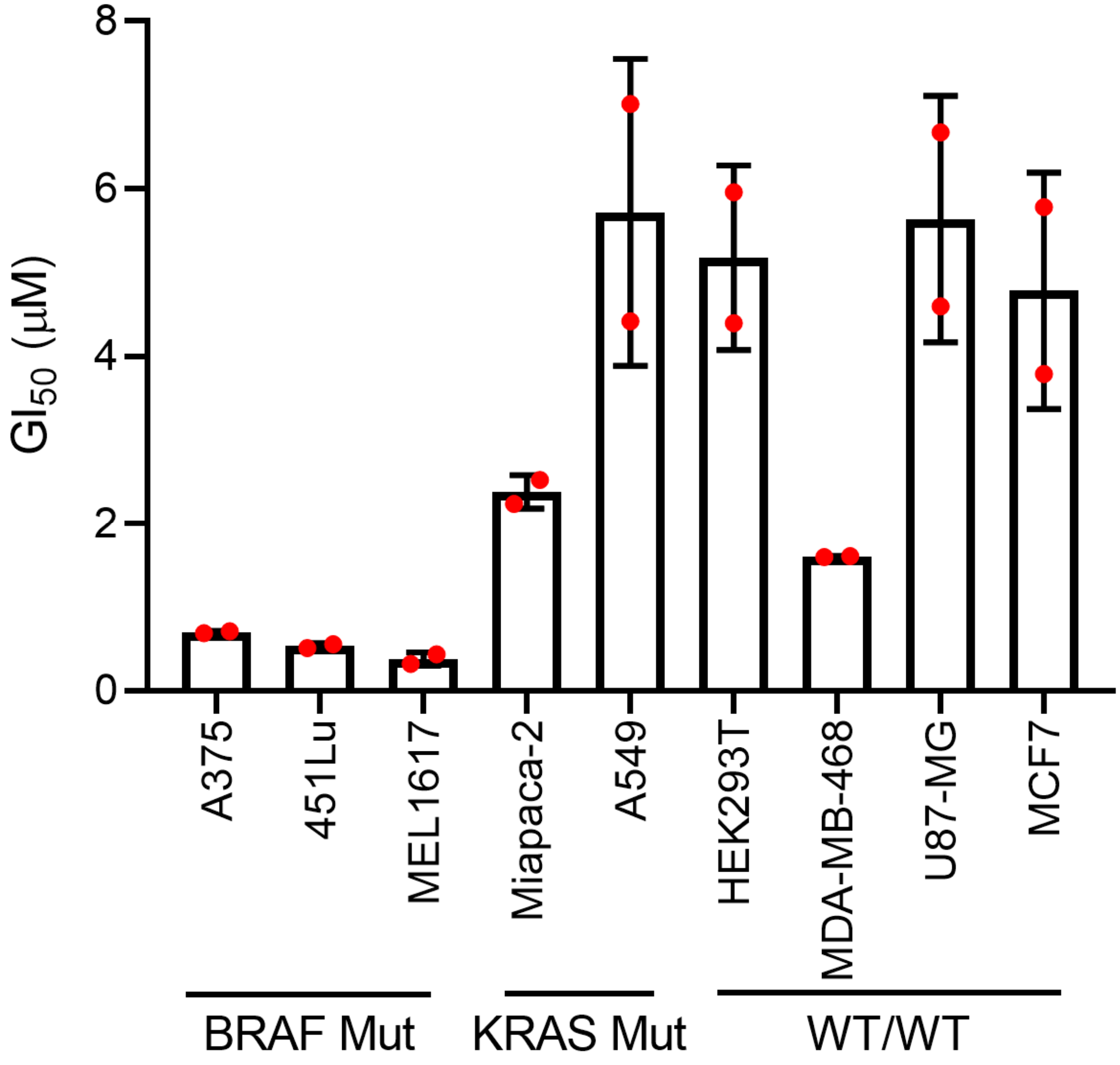

Supplementary Fig. 24 BI-78D3 activity towards various BRAF/KRAS mutant and wild-type cancer cell lines. Viability was determined after 48hrs of treatment using MTS assay (Promega). The Y-axis represents BI-78D3 GI_{50} (The concentration that resulted in 50% Growth inhibition) versus tumor cell line on the X-axis. Data show the average from six independent experiments; Error bars ±SD.

Fig 4b.

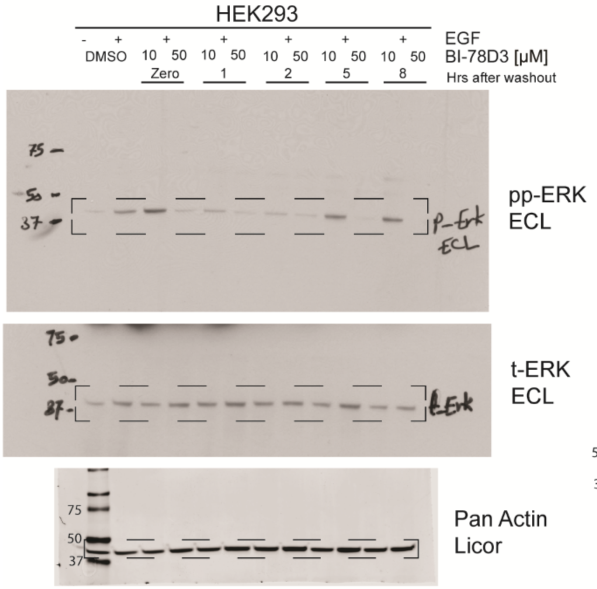

Fig 4c.

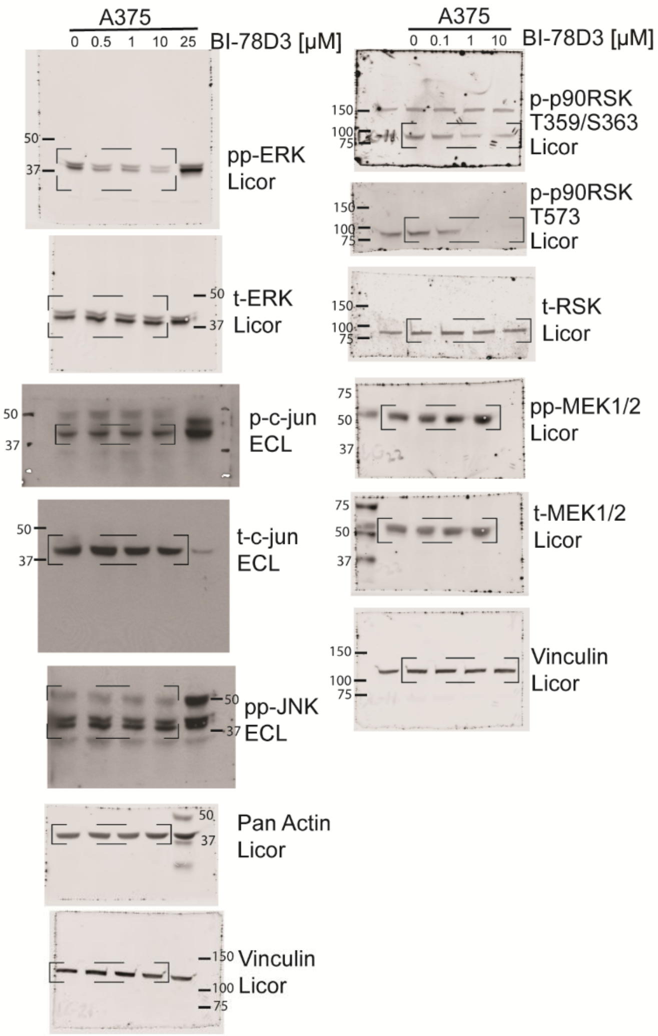

Fig 5b.

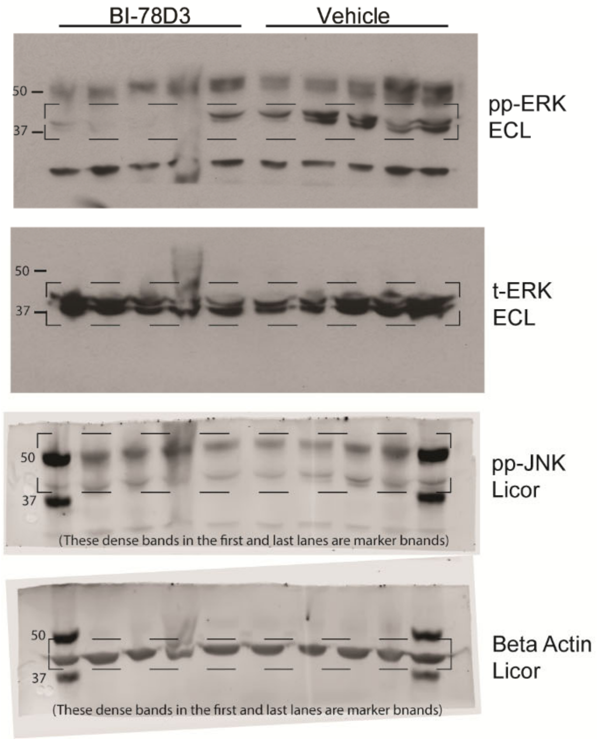

Supplementary Fig. 25 Original uncropped images of western blots of Figs. 4b, 4c and 5b.

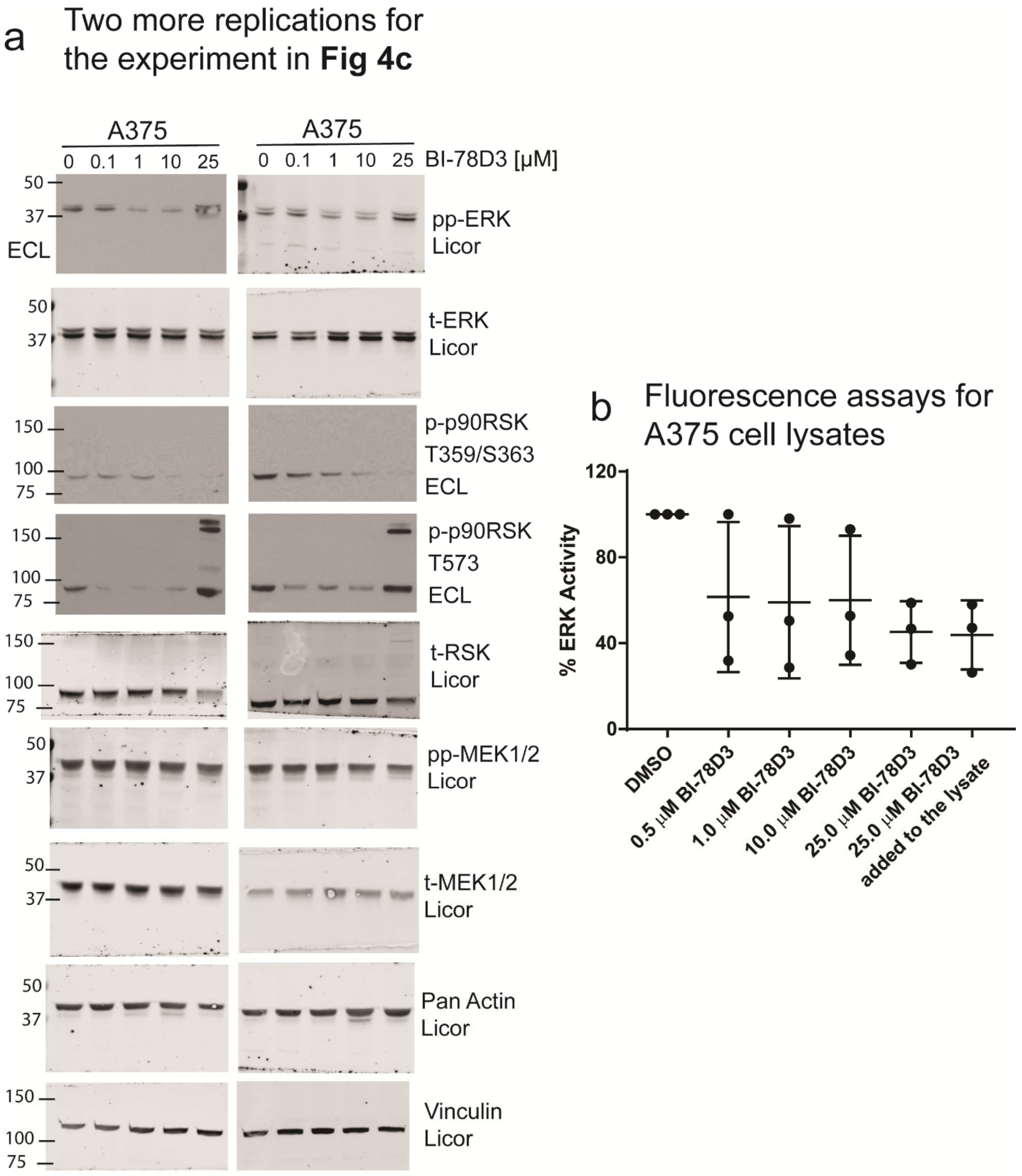

Supplementary Fig. 26 a Original uncropped images of western blots for two more replicates of the experiment mentioned in Fig. 4c and Supplementary Fig. 25. **b** Fluorescence assays for A375 cell lysates. In this experiment, 1.5 million A375 cells were seeded in 60 mm dish, serum starved

overnight, treated with BI-78D3 for 1 hour in serum free media, followed by inhibitor washout for 2 hours in the same media. Cells were then stimulated with EGF for 30 minutes in full media. The assay was done immediately after cell lysis**.** The protocols of cell lysis and fluorescence assay are mentioned in details in the methods section.

Fig 6i.

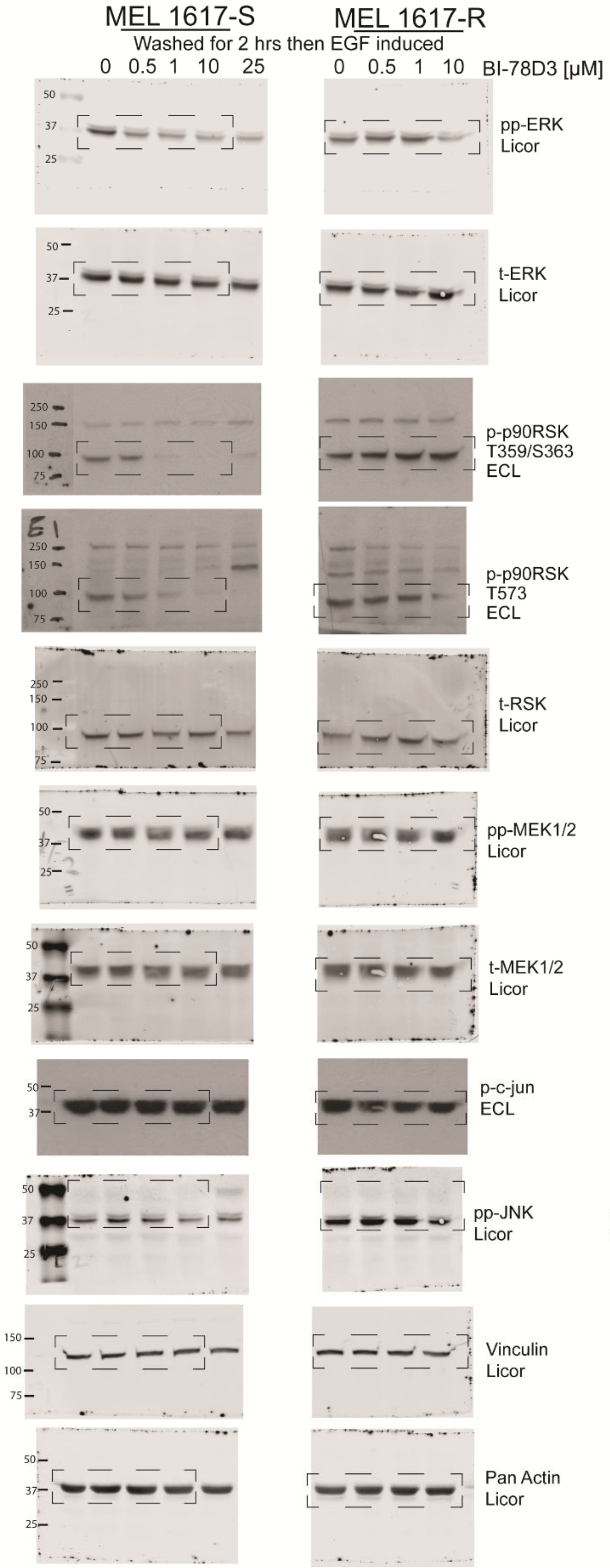

Supplementary Figure 16b.

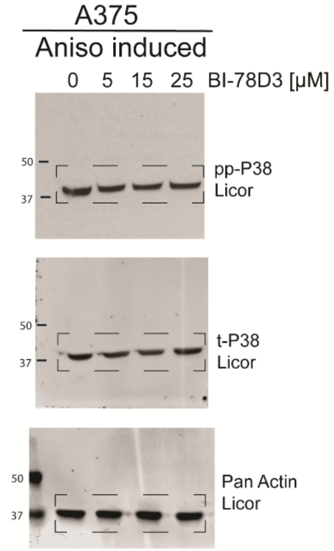

Supplementary Figure 16c.

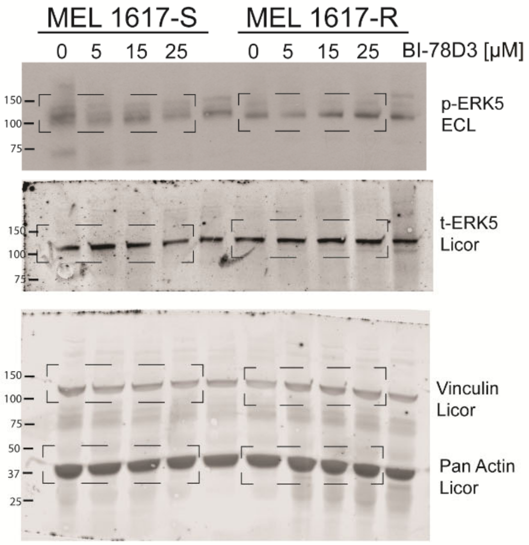

Supplementary Fig. 27 Original uncropped images of western blots of Fig. 6i, supplementary Fig. 16b and supplementary Fig. 16c

Supplementary Figure 19.

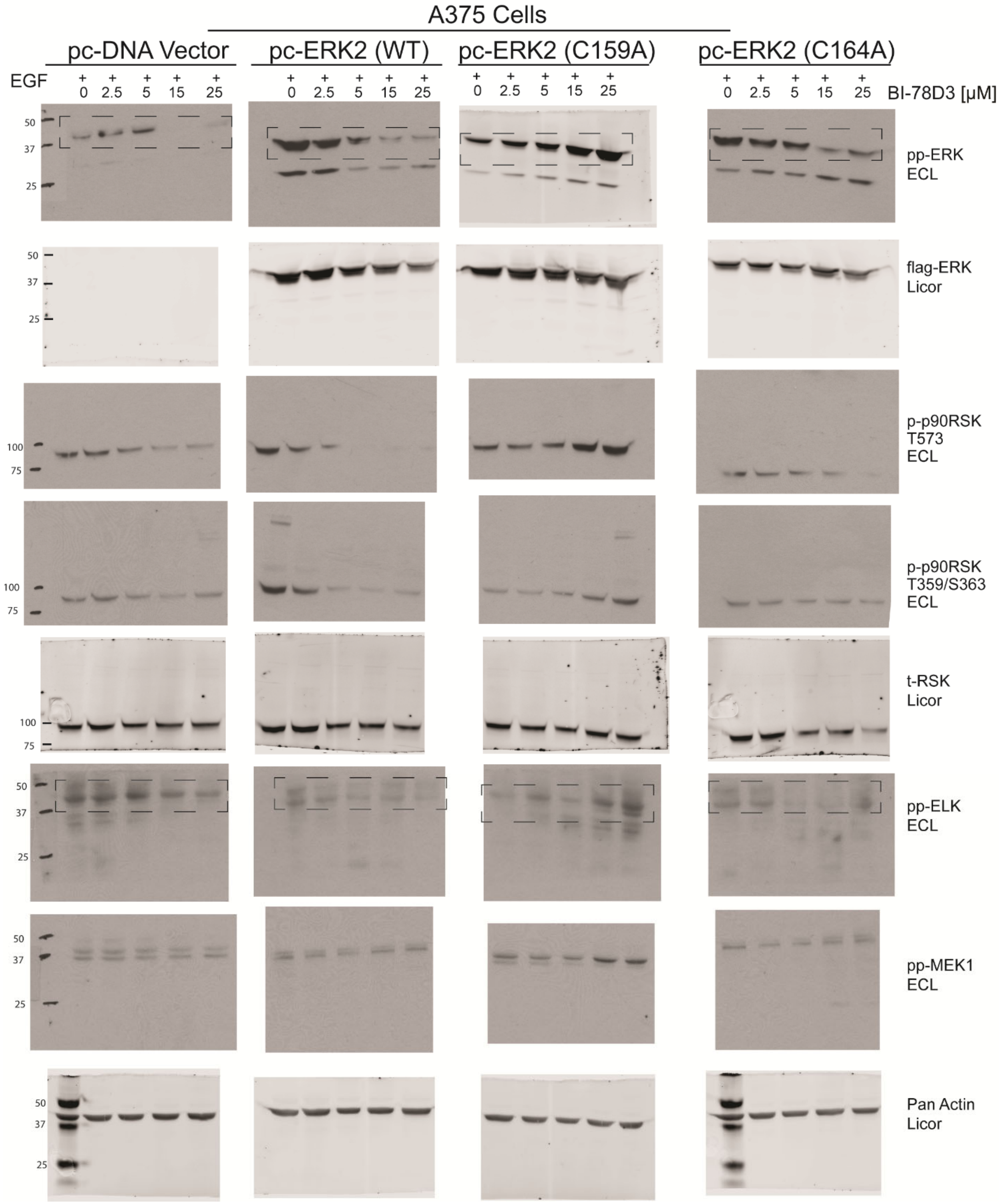

Supplementary Fig. 28 Original uncropped images of western blots of supplementary Fig. 19.

Supplementary Figure 22.

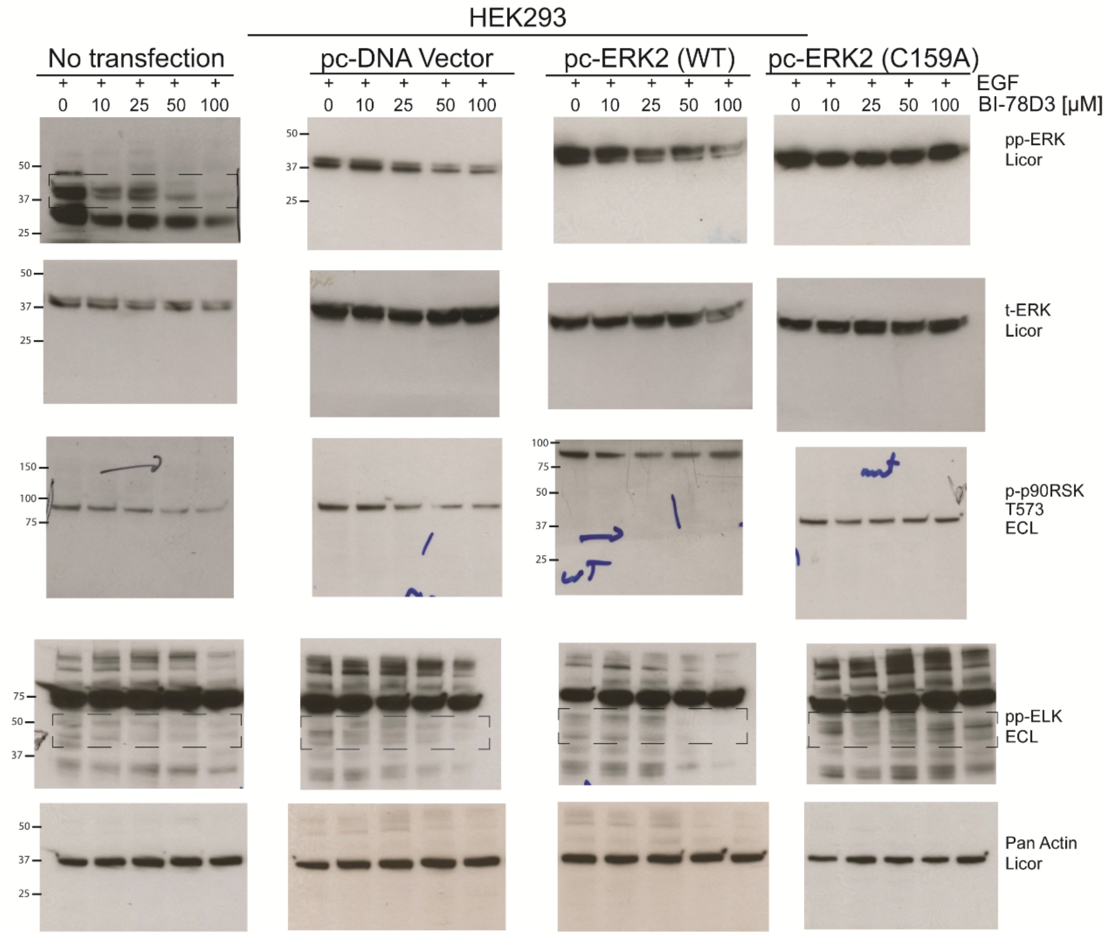

Supplementary Fig. 29 Original uncropped images of western blots of supplementary Fig. 22.

Supplementary Methods:

Synthesis and Characterization of Methyl 2-((4-(2,3-dihydrobenzo[b][1,4]dioxin-6-yl)-5-oxo-4,5-dihydro-1H-1,2,4-triazol-3-yl)thio)thiazole-5-carboxylate (Con-1) (**4**).[8]

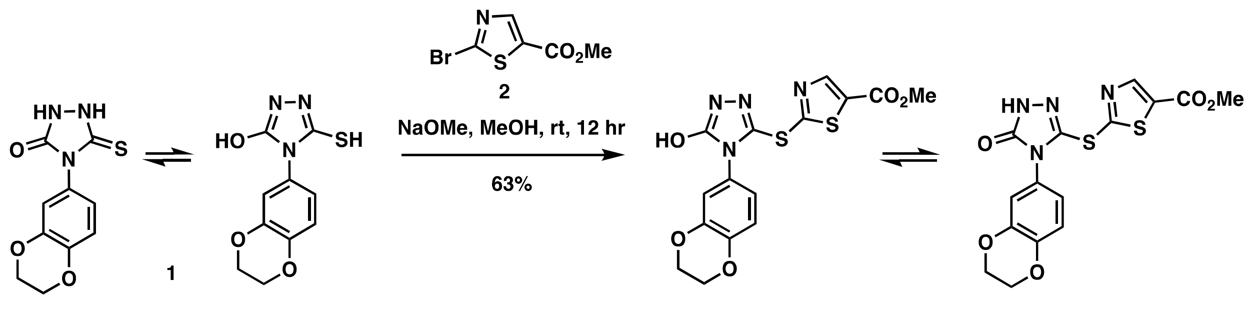

To a solution of 4-(2,3-dihydrobenzo[b][1,4]dioxin-6-yl)-5-mercapto-4H-1,2,4-triazol-3-ol 1 (100 mg, 0.398 mmol) in MeOH (2 mL) was added NaOMe (22 mg, 0.398 mmol) at room temperature. The reaction mixture was stirred for 5 min, and methyl 2-bromothiazole-5-carboxylate 2 (88 mg, 0.398 mmol) was added and then stirring continued overnight. The reaction mixture was acidified with 1 N HCl, and the resulting precipitate was collected by filtration in vacuo and washed obtained cake with water (3 x 30 mL), and 10% ethyl acetate in hexanes (2 x 30 mL) to afford crude Con-1. The crude was chromatographed using silica gel (1–3% MeOH in dichloromethane) to obtain pure Con-1 as a solid (98 mg, 63%). ^{1}H NMR (400 MHz, DMSO-d6) δ 12.70 (s, 1H), 8.32 (s, 1H), 6.92–6.871 (m, 2H), 6.79–6.77ff (m, 1H), 4.25 (m, 4H), 3.82 (s, 3H); ^{13}C NMR (100 MHz, DMSO-d6) δ 166.4, 160.2, 153.8, 148.5, 144.0, 143.3, 137.4, 130.2, 124.9, 120.5, 117.2, 116.5, 64.1, 64.0, 52.7. HRMS m/z found 393.0329, calculated for $C_{15}H_{12}N_4O_5S_2$ [M+H]+ 393.0322.

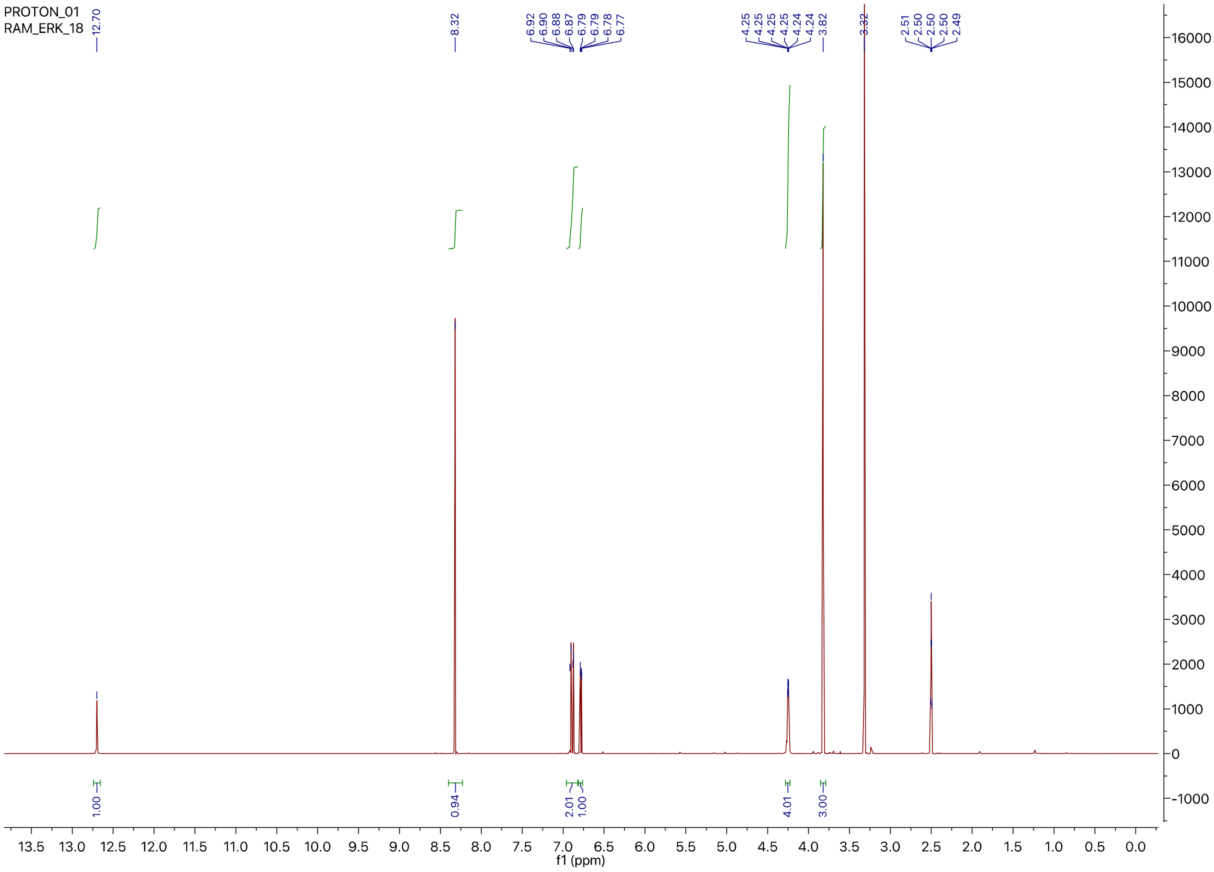

Supplementary Fig. 30 ^{1}H NMRs of Con-1 (**4**).

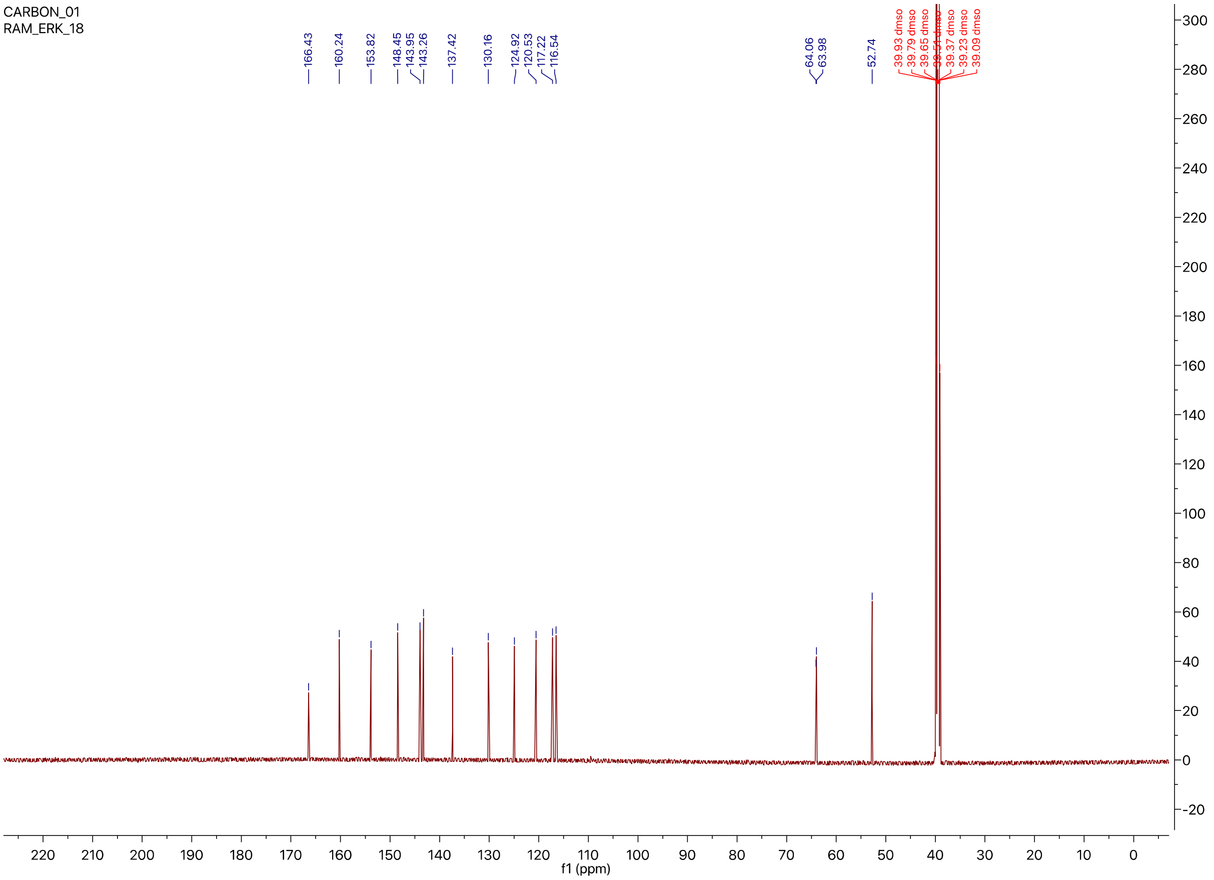

Supplementary Fig. 31 ^{13}C NMR of Con-1 (**4**).

Target Compound Screening Report

**Data File**	MSF16-3603(RAM-ERK-18)_hrESIpos1.d	**Sample Name**	3603(RAM-ERK-18)	**Comment**	3603(RAM-ERK-18)
**Position**	P1-B4	**Instrument Name**	Instrument 1	**User Name**	
**Acq Method**	pos.m	**Acquired Time**	10/20/2016 4:34:18 PM	**DA Method**	KS.m

MS Zoomed Spectrum

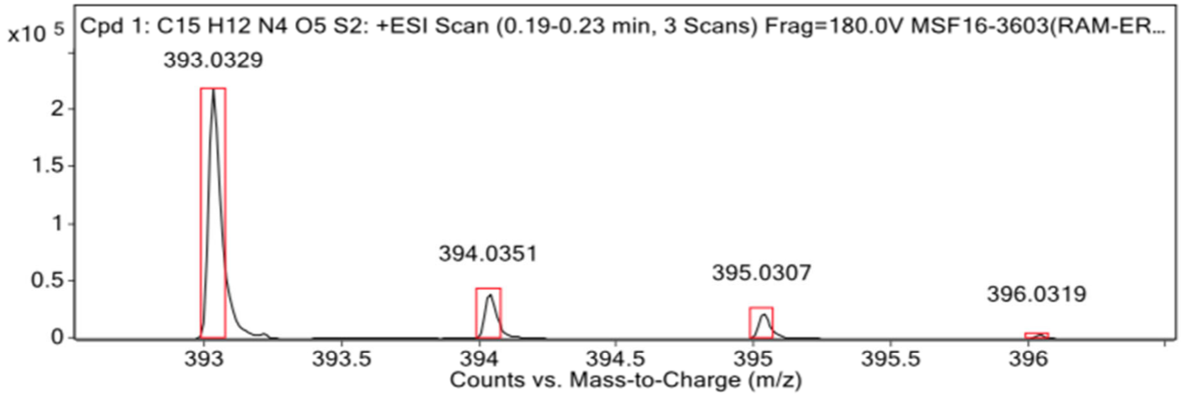

MS Spectrum Peak List

Obs. m/z	Calc. m/z	Charge	Abund	Formula	Ion/Isotope	Tgt Mass Error (ppm)
393.03290	393.03220	1	219089.25	C15H12N4O5S2	(M+H)+	-1.92
394.03510	394.03480	1	39590.41	C15H12N4O5S2	(M+H)+	-0.81
395.03070	395.03020	1	22786.88	C15H12N4O5S2	(M+H)+	-1.36
396.03190	396.03200	1	4054.9	C15H12N4O5S2	(M+H)+	0.38
397.03410	397.02940	1	887.8	C15H12N4O5S2	(M+H)+	-11.73
415.01530			557019.07			

--- End Of Report ---

Supplementary Fig. 32 High resolution MS of Con-1 (**4**).

Molecular Dynamics Simulations. An initial complex was generated by docking the compound into crystal structure 4ERK using GOLD.[9] A distance constraint of between 1.5 and 2.5 angstroms was placed between the sulfur of C159 and the appropriate carbon atom of the ligand. All simulations were carried out with a 3 fs time step using the AMOEBA force field,[10] runs on GPUs using Tinker-OpenMM.[11] Protein parameters have defaulted ameobapro13. A distant constraint of 5 kcal A^{-2} and distance between 1.5 and 2.0 A was used to sample the pre-reaction complex between the sulfur of C159 and the carbon atom. AMOEBA parameters of the compound were generated using poltype.[12] A solvated model (100 Å on each side) of the complex was generated using the structure generated by docking. The resulting complex was heated for from 25 K to 298 K (increasing 25 degrees every 100 ps). After reaching 298 K, the complex was simulated for 100 ns, and the final frame was analyzed.

Supplementary References

1 Pearson, G. *et al.* Mitogen-activated protein (MAP) kinase pathways: regulation and physiological functions. *Endocr Rev* **22**, 153-183, doi:10.1210/edrv.22.2.0428 (2001).

2 Shaul, Y. D. & Seger, R. The MEK/ERK cascade: from signaling specificity to diverse functions. *Biochim Biophys Acta* **1773**, 1213-1226, doi:10.1016/j.bbamcr.2006.10.005 (2007).

3 Albeck, J. G., Mills, G. B. & Brugge, J. S. Frequency-modulated pulses of ERK activity transmit quantitative proliferation signals. *Mol Cell* **49**, 249-261, doi:10.1016/j.molcel.2012.11.002 (2013).

4 Canfield, R. E. & Liu, A. K. The Disulfide Bonds of Egg White Lysozyme (Muramidase). *J Biol Chem* **240**, 1997-2002 (1965).

5 Muta, Y. *et al.* Composite regulation of ERK activity dynamics underlying tumour-specific traits in the intestine. *Nat Commun* **9**, 2174, doi:10.1038/s41467-018-04527-8 (2018).

6 Davies, H. *et al.* Mutations of the BRAF gene in human cancer. *Nature* **417**, 949-954, doi:10.1038/nature00766 (2002).

7 Chapman, P. B. *et al.* Improved survival with vemurafenib in melanoma with BRAF V600E mutation. *N Engl J Med* **364**, 2507-2516, doi:10.1056/NEJMoa1103782 (2011).

8 Flaherty, K. T. *et al.* Combined BRAF and MEK Inhibition in Melanoma with BRAF V600 Mutations. *N Engl J Med*, doi:10.1056/NEJMoa1210093 (2012).

9 Flaherty, K. T. *et al.* Inhibition of mutated, activated BRAF in metastatic melanoma. *N Engl J Med* **363**, 809-819, doi:10.1056/NEJMoa1002011 (2010).

10 Long, G. V. *et al.* Dabrafenib in patients with Val600Glu or Val600Lys BRAF-mutant melanoma metastatic to the brain (BREAK-MB): a multicentre, open-label, phase 2 trial. *The Lancet Oncology* **13**, 1087-1095, doi:10.1016/S1470-2045(12)70431-X (2012).

11 Welsh, S. J., Rizos, H., Scolyer, R. A. & Long, G. V. Resistance to combination BRAF and MEK inhibition in metastatic melanoma: Where to next? *Eur J Cancer* **62**, 76-85, doi:10.1016/j.ejca.2016.04.005 (2016).

12 Amaral, T. *et al.* MAPK pathway in melanoma part II-secondary and adaptive resistance mechanisms to BRAF inhibition. *Eur J Cancer* **73**, 93-101, doi:10.1016/j.ejca.2016.12.012 (2017).
