# Supplementary material for: Modulating multi-functional ERK complexes by covalent targeting of a recruitment site in vivo
Source: Nat Commun. 2019 Nov 19;10:5232. doi: 10.1038/s41467-019-12996-8 (PMC6863825; doi:10.1038/s41467-019-12996-8)
Supplement: Supplementary file 3 — Reporting Summary [file 41467_2019_12996_MOESM3_ESM.pdf]

nature research

Corresponding author(s): Kevin Dalby, PhD

Reporting Summary

Nature Research wishes to improve the reproducibility of the work that we publish. This form provides structure for consistency and transparency in reporting. For further information on Nature Research policies, see Authors & Referees and the Editorial Policy Checklist.

Statistical parameters

When statistical analyses are reported, confirm that the following items are present in the relevant location (e.g. figure legend, table legend, main text, or Methods section).

n/a	Confirmed	
☐	☒	The exact sample size (n) for each experimental group/condition, given as a discrete number and unit of measurement
☐	☒	An indication of whether measurements were taken from distinct samples or whether the same sample was measured repeatedly
☐	☒	The statistical test(s) used AND whether they are one- or two-sided<br>*Only common tests should be described solely by name; describe more complex techniques in the Methods section.*
☒	☐	A description of all covariates tested
☐	☒	A description of any assumptions or corrections, such as tests of normality and adjustment for multiple comparisons
☐	☒	A full description of the statistics including central tendency (e.g. means) or other basic estimates (e.g. regression coefficient) AND variation (e.g. standard deviation) or associated estimates of uncertainty (e.g. confidence intervals)
☐	☒	For null hypothesis testing, the test statistic (e.g. F, t, r) with confidence intervals, effect sizes, degrees of freedom and P value noted<br>*Give P values as exact values whenever suitable.*
☒	☐	For Bayesian analysis, information on the choice of priors and Markov chain Monte Carlo settings
☒	☐	For hierarchical and complex designs, identification of the appropriate level for tests and full reporting of outcomes
☒	☐	Estimates of effect sizes (e.g. Cohen's d, Pearson's r), indicating how they were calculated
☐	☒	Clearly defined error bars<br>*State explicitly what error bars represent (e.g. SD, SE, CI)*

Our web collection on statistics for biologists may be useful.

Software and code

Policy information about availability of computer code

Data collection	For collection of flow cytometry data, BD FACSDiva v6.1.3. software was used. UCSF Chimera software was used to generate the molecular models and protein alignment images. Tinker-OpenMM, ameobapro13 and poltype softwares were used to perform the molecular dynamics simulations. Image Studio Lite Ver 5.2 was used to generate the western blotting images using the Licor Odyssey instrument. IncuCyte ZOOM software was used to collect and analyze the IncuCyte microscope data.
Data analysis	Open CFU software were employed to analyse the colonies formation data. GrapPad Prism 7 software and Kaleidagraph software were used for enzyme kinetics studies and statistical analysis. The cell cycle analysis was performed using FlowJo v10 (Tree Star, Inc., Ashland, OR, USA) software, ModFit 3.0 (Verity) software. The ImageJ software was employed to analyze the confocal microscope images.

For manuscripts utilizing custom algorithms or software that are central to the research but not yet described in published literature, software must be made available to editors/reviewers upon request. We strongly encourage code deposition in a community repository (e.g. GitHub). See the Nature Research guidelines for submitting code & software for further information.

Data

Policy information about availability of data

All manuscripts must include a data availability statement. This statement should provide the following information, where applicable:

- Accession codes, unique identifiers, or web links for publicly available datasets
- A list of figures that have associated raw data
- A description of any restrictions on data availability

All the data supporting the findings of this study are available in this manuscript and its supplementary information.

Field-specific reporting

Please select the best fit for your research. If you are not sure, read the appropriate sections before making your selection.

☒ Life sciences ☐ Behavioural & social sciences ☐ Ecological, evolutionary & environmental sciences

For a reference copy of the document with all sections, see nature.com/authors/policies/ReportingSummary-flat.pdf

Life sciences study design

All studies must disclose on these points even when the disclosure is negative.

Sample size	For all experiments at least three independent replicates were taken for each data point, unless otherwise indicated.
Data exclusions	No data were excluded from the datasets.
Replication	Two independent experiments at least were repeated with similar results, the number of replicates are mentioned in the figure legends and/ or the methods section.
Randomization	The animals were randomly assigned to different experimental groups.<br>The position of samples on multiwell plates were different between individual experiments to minimize the systematic errors.
Blinding	No blinding.

Reporting for specific materials, systems and methods

Materials & experimental systems

n/a	Involved in the study	
☒	☐	Unique biological materials
☐	☒	Antibodies
☐	☒	Eukaryotic cell lines
☒	☐	Palaeontology
☐	☒	Animals and other organisms
☒	☐	Human research participants

Methods

n/a	Involved in the study	
☒	☐	ChIP-seq
☐	☒	Flow cytometry
☒	☐	MRI-based neuroimaging

Antibodies

Antibodies used	ANTI-FLAG® M2-FITC antibody produced in mouse (Cat # F4049, Sigma); 1:2000 anti-phospho-p44/42 MAPK (ERK1/2) (Thr202/ Tyr204) (E10) mouse mAb (Cat # 9106, Cell Signaling Technology); 1:1000 anti p44/42 MAPK (ERK1/2) (137F5) rabbit mAb (Cat # 4695, Cell Signaling Technology); 1:1000 anti DYKDDDDK Tag (9A3) mouse mAb (Cat # 8146, Cell Signaling Technology); 1:1000 anti-phospho-p90RSK (Thr573) rabbit polyclonal Abs (Cat # 9346, Cell Signaling Technology); 1:1000 anti-phospho-p90RSK (Thr359/Thr363) rabbit polyclonal Abs (Cat # 9344, Cell Signaling Technology); 1:1000 anti-RSK1/RSK2/RSK3 (32D7) Rabbit mAb (Cat # 9355, Cell Signaling Technology); 1:500 anti-phospho-Elk-1 (Ser-383) rabbit polyclonal IgG (Invitrogen); 1:1000 anti-phospho-MEK1/2 (Ser217/221) rabbit mAb (Cat # 9154, Cell Signaling Technology); 1:1000 anti-MEK1/2 (L38C12) mouse mAb (Cat # 4694, Cell Signaling Technology); 1:2000 anti-phospho-SAPK/JNK (Thr183/Tyr185) (G9) mouse mAb (Cat # 9255, Cell Signaling Technology); 1:2000 anti-JNK2 (56G8) rabbit mAb (Cat # 9258, Cell Signaling Technology); 1:10,000 anti-phospho-c-Jun (Ser-63), clone Y172 rabbit mAb (Cat # 04-212, Millipore) or 1:1000 anti-phospho-c-Jun (Ser-63) II rabbit polyclonal Ab (Cat # 9261, Cell Signaling Technology); 1:1000 anti-c-Jun (60A8) rabbit mAb (Cat # 9165, Cell Signaling Technology); 1:10,000 anti-phospho-p38α (Thr180/Tyr182), clone 8.78.8 rabbit mAb (Cat # 05-1059, Millipore) or 1:1000 anti-phospho-p38 MAPK (Thr180/

	Tyr182) (D3F9) XP rabbit mAb (Cat # 4511, Cell Signaling Technology); 1:1000 anti-p38 MAPK (D13E1) XP rabbit mAb (Cat # 8690, Cell Signaling Technology); 1:1000 anti-phospho-BMK1/Erk5 (Thr218/Tyr220) rabbit polyclonal Ab (Cat # 07-507, Millipore); 1:1000 anti-Erk5 rabbit polyclonal Ab (Cat # 3372, Cell Signaling Technology); 1/2000 anti-Vinculin (E1E9V) XP rabbit mAb (Cat # 13901, Cell Signaling Technology) and 1:5000 anti-actin, clone 4 mouse mAb (Cat # MAB1501R, Millipore). Secondary anti-rabbit (Bio-Rad) or anti-mouse (Bio-Rad) horseradish peroxidase-conjugated secondary antibodies or Li-Cor: IRDye 680RD goat anti-rabbit IgG and IRDye 800CW goat anti-mouse IgG.
Validation	Both primary and secondary antibodies are used on a weekly basis in our lab. Additional information on the antibodies are available on the manufacture's websites.

Eukaryotic cell lines

Policy information about cell lines

Cell line source(s)	Human Embryonic Kidney cell line (HEK-293T-ATCC CRL-3216) and A375 melanoma cell line (ATCC-CRL-1619) were purchased from the American type culture collection (ATCC; Manassas, VA). 451 Lu and MEL 1617 melanoma cell lines were provided by Prof. Kenneth Tsai, H. Lee Moffitt Cancer Center & Research Institute, Florida, USA. Human pancreas ductal adenocarcinoma cell line (MIA PaCa-2 – ATCC CRL-1420), human breast adenocarcinoma cell line (MCF7-ATCC HTB-22) and human non-small cell lung cancer cell line (A549-ATCC CCL-185) were purchased from the American type culture collection (ATCC; Manassas, VA). Human Glioma cell line (U87-MG-ATCC HTB-14) was obtained from the Neurosurgery Tissue Bank, University of California, San Francisco, USA. Human breast adenocarcinoma cell line (MDA-MB-468 – ATCC HTB-132) was a gift from Dr. Chandra Bartholomeusz, The University of Texas MD Anderson Cancer Center, Houston, TX, USA.
Authentication	Cells that were purchased from ATCC were authenticated by the ATCC using short, tandem-repeat profiling. Cells that were provided by research groups in The University of Texas MD Anderson Cancer Center, were validated by STR DNA fingerprinting using the AmpFℓSTR Identifiler kit according to manufacturer instructions (Applied Biosystems, Grand Island, NY). The rest of the cell lines were verified using Human 9-Marker STR Profile and Interspecies Contamination Test - IDEXX BioResearch
Mycoplasma contamination	Mycoplasma tests were performed monthly using MycoAlert™ Mycoplasma Detection Kit (Lonza). No infection has been detected during experiments presented in this study.
Commonly misidentified lines (See ICLAC register)	No commonly misidentified cell lines were used

Animals and other organisms

Policy information about studies involving animals; ARRIVE guidelines recommended for reporting animal research

Laboratory animals	Five-week-old, Inbred, athymic nude Mus musculus (Foxn1 (nu) homozygous strains).
Wild animals	The study did not involve wild animals.
Field-collected samples	The study did not involve samples collected from the field.

Flow Cytometry

Plots

Confirm that:

- [x] The axis labels state the marker and fluorochrome used (e.g. CD4-FITC).
- [x] The axis scales are clearly visible. Include numbers along axes only for bottom left plot of group (a 'group' is an analysis of identical markers).
- [ ] All plots are contour plots with outliers or pseudocolor plots.
- [x] A numerical value for number of cells or percentage (with statistics) is provided.

Methodology

Sample preparation	Cells were harvested and washed twice with phosphate buffered saline (PBS) and fixed in ice cold 70% ethanol overnight at -20 ° C. The next day cells were washed ×2 with PBS and re-suspended in propidium podide (PI)/RNase staining solution (cell signaling Cat # 4087) for 30 minutes at room temperature and protected from light.
Instrument	BD LSRFortessa SORP Flow Cytometer
Software	Sample acquisition and analysis is done using FACSDiva v6.1.3. Further analysis were done on FlowJo v10 (Tree Star, Inc., Ashland, OR, USA) software and ModFit 3.0 (Verity) software
Cell population abundance	100%
Gating strategy	Single cell nuclei were identified based on FSC-A and FSC-H; cell debris was excluded from the analysis.

Gating strategy

The event rate was adjusted to <500/second.

☐ Tick this box to confirm that a figure exemplifying the gating strategy is provided in the Supplementary Information.
